# The effect of cCMP and cUMP on growth of *Pseudomonas aeruginosa*

**DOI:** 10.3389/fmicb.2025.1675794

**Published:** 2025-09-16

**Authors:** Christoph Risser, Justin Rothschuh, Heike Bähre, Detlef Neumann, Roland Seifert, Bastian Schirmer

**Affiliations:** ^1^Hannover Medical School, Institute of Pharmacology, Hannover, Germany; ^2^Hannover Medical School, Research Core Unit Metabolomics, Hannover, Germany

**Keywords:** cyclic nucleotides, 5’-cCMP, 5’-cUMP, *Pseudomonas aeruginosa*, biofilm

## Abstract

The aim of this study was to screen for possible biological effects of the non-canonical nucleotides 3’,5’-cyclic uridine monophosphate (cUMP) and 3’,5’-cyclic cytidine monophosphate (cCMP) on Pseudomonas aeruginosa beyond the already reported function in the bacterial pyrimidine cyclase system for antiphage resistance (Pycsar). Since endogenously synthesized cCMP was detected in growing bacterial cultures and the cCMP concentration was higher when nutrients became more restricted, we hypothesized that a membrane-permeable analog of cCMP added to growing cultures alters growth kinetics. Indeed, when growing in a nutrient-scarce minimum salt medium, the cCMP analog acetoxymethyl-cCMP led to a dose-dependent growth lag, whereas neither its native counterpart nor cAMP, cGMP, or cUMP induced such a lag. This inhibitory effect on growth translated into a sensitizing effect against the antibacterial drugs azithromycin and, very pronounced, against gentamicin. Since *P. aeruginosa* is one of the most common opportunistic pathogens, improving antibacterial drug therapies is of high interest and may be a promising future research area. Exposure of bacterial cultures to native cUMP led to induction of biofilm formation, which was paralleled by an increase in c-di-GMP synthesis and generation of the Quorum sensing metabolites pqs and hhq. Since biofilm formation is another key feature of Pseudomonas aeruginosa to evade the host’s immune system, targeting cyclic UMP concentrations during infections may also be of therapeutic relevance. In summary, our findings corroborate the need for further research on the 3’,5’-cyclic pyrimidine nucleotides in bacteria beyond their established function in the Pycsar anti-phage defense system.

## 1 Introduction

The gram-negative rod bacterium *Pseudomonas aeruginosa* (*P. aeruginosa*) is a frequent cause of chronic respiratory tract infections in patients with cystic fibrosis (CF), bronchiectasis, or chronic obstructive pulmonary disease (COPD) (Valderrey et al., 2010). It is one of the most common opportunistic pathogens in nosocomial infections such as ventilation-associated pneumonia (Fujitani et al., 2011; Barbier et al., 2013). Due to its intrinsic resistance to several classes of antibacterial drugs (e.g., β-lactams, aminoglycosides, and quinolones), a deeper understanding of the communication and behavior of *P. aeruginosa* is needed to identify new therapeutic targets (World Health Organization, 2017; Pang et al., 2019).

Beyond intrinsic resistance, a key feature of *P. aeruginosa* is its ability to form biofilms (Thi et al., 2020). Biofilm formation is largely regulated by the common bacterial second messenger bis-(3’,5’)-cyclic guanosine monophosphate (c-di-GMP), which is synthesized by diguanylyl cyclases (DGC) and degraded by specific phosphodiesterases (PDEs) (Jenal et al., 2017; Bhasme et al., 2020). Increasing concentrations of c-di-GMP are associated with decreased motility and decreased metabolism, but higher secretion and production of extracellular polysaccharides such as alginate, Pel, and Psl (Lichtenberg et al., 2022).

The three Quorum sensing (QS) pathways las, rhl, and pqs also regulate biofilm establishment (Thi et al., 2020; Chadha et al., 2022). The QS signal molecule classes N-acyl-L-homoserine lactones (AHLs) and 2-alkyl 4-(1H)-quinolones (AQs) affect motility, biofilm formation, and expression of virulence factors (Rutherford and Bassler, 2012; Dubern et al., 2023). The two signal molecules 2-heptyl-4-hydroxyquinolone (HHQ) and pseudomonas quinolone signal (PQS) from the pqs pathway are highly associated with biofilm formation (Williams and Cámara, 2009). At present, the interaction between the QS pathways and c-di-GMP is still not fully understood (Srivastava and Waters, 2012). Proteins of the QS pathway, e.g., PqsE, can bind to the PDE ProE, modulate c-di-GMP concentration and elicit a specific reaction (Feng et al., 2025). Additionally, it is assumed that there are other protein-protein interactions between QS proteins, the DGCs and the PDEs that might provide bilateral exchange processes (Chua et al., 2017). This is supported by findings in other gram-negative bacteria, where QS and c-di-GMP are connected by transcription factors (Yang et al., 2017; Hochstrasser and Hilbi, 2020). Regardless of the pathway that led to biofilm formation, the bacteria can evade the human immune system and thus complicate therapeutic treatment of infections being protected by the biofilm matrix (Lewis, 2001).

Even before the “era” of c-di-GMP, 3’,5’-cyclic nucleotides have been described as messengers in cellular regulation. In bacterial cells, the non-canonical nucleotides 3’,5’-cyclic uridine monophosphate (cUMP) and 3’,5’-cyclic cytidine monophosphate (cCMP) have only recently moved into the focus of many scientists. As discovered by Tal et al. (2021), the uridylyl cyclase PycC in *P. aeruginosa* is involved in anti-phage defense, as a part of the bacterial pyrimidine cyclase system for antiphage resistance (Pycsar) immune system (Tal et al., 2021; Seifert and Schirmer, 2022). In case of a phage infection, PycC is activated and the intrabacterial cUMP concentration rises thus activating the cUMP-specific effector PycTIR, which acts as NADase leading to a cellular energy deficiency (Tal et al., 2021). Consequently, the bacterial metabolism, including virus replication in the host bacteria, decreases preventing spread of the phage infection to the “peer” bacteria.

In other bacterial species, such as *Staphylococcus aureus* and *Escherichia coli*, the transmembrane protein PycTM has been identified as effector of the Pycsar system (Tal et al., 2021). Binding of cCMP to PycTM leads to membrane impairment and subsequent cell damage, impeding the replication of phages in the infected bacteria. No specific cytidylyl cyclase nor a specific cCMP effector, such as PycTM, could be detected in *P. aeruginosa* until now, but the promiscuous nucleotidyl cyclase ExoY is expressed by the bacteria as one of the four effector proteins injected by the type-3-secretion system (T3SS) into host cells. After binding to host actin, ExoY produces mainly cGMP/cUMP and smaller amounts of cAMP and cCMP (Beckert et al., 2014; Belyy et al., 2018; Kloth et al., 2018). In an acute mouse lung infection model, strains with overexpressed ExoY have shown an increase in severity of infection (Kloth et al., 2018). These findings suggest biological functions of cCMP and cUMP that might go beyond the realm of bacteria and include eukaryotic cells (Bähre et al., 2015).

Here, we report possible biological effects of cCMP and cUMP on *P. aeruginosa* concerning growth, resistance to antibacterial drugs, biofilm formation, and quorum sensing. Since both cUMP and cCMP have deleterious effects on the bacteria in the known bacterial Pycsar systems, we hypothesize that, independent of the presence of a functional Pycsar system, cCMP and cUMP will impede bacterial growth or induce protective behavior.

## 2 Materials and methods

Cyclic nucleotides and their respective membrane-permeable acetoxymethyl ester analogs (AM) were supplied by Biolog Life Science Institute GmbH & Co. KG, Bremen, Germany. All other reagents were supplied by Sigma-Aldrich, if not stated otherwise.

### 2.1 Bacterial strains used in this study

For the measurement of endogenous 3’,5’-cyclic nucleotides (cNMP) concentrations, *P. aeruginosa* strains PA14 and PAO1 have been used, all other experiments have been performed with *P. aeruginosa* strain PA14, only. Both strains are genomically devoid of the Pycsar system components PaPycC (cUMP generator) and PaPycTIR (cUMP effector).

### 2.2 Bacterial culture for measurement of intracellular cNMP concentrations

The bacteria were cultured at 37 °C shaking (160 rpm), harvested by centrifugation (2,500 × *g*, 20 min, 4 °C), washed with medium and then re-suspended with medium. Two different media were used: First, Vogel-Bonner (VB) medium (10-fold, pH 7) consisting of 8, 11 × 10^–3^ M MgSO_4_⋅7 H_2_O, 0.1 M citric acid, 0.57 M K_2_HPO_4_, 0.13 M NaNH_4_HPO_4_⋅4 H_2_O, and second, Luria-Bertani (LB) medium (pH 7; Difco™ LB Broth, BD, Sparks, United States). Optic density of the bacteria culture was measured at 600 nm using an Ultrospec 10 Cell Density Meter (GE Healthcare, Amersham, United States). For cNMP stimulating assay cCMP or cUMP was added to the medium up to a concentration of 1 μM. As a control, bacteria cultured without adding cNMPs were used.

### 2.3 Sample preparation for mass spectrometric measurement of cNMPs

The extraction of cyclic nucleotides from bacterial cultures was based on earlier descriptions (Rabinowitz and Kimball, 2007; Spangler et al., 2010) and was slightly adapted for cNMPs as follows. cNMP analysis of harvested bacteria were carried out by adding 300 μl of a mixture of acetonitrile/methanol/water (2/2/1, v/v/v) containing 25 ng/ml of the internal standard (tenofovir), following 15 min of cooling and incubation. For phosphodiesterase inactivation, samples were heated for 15 min at 95 °C. Thereafter samples were centrifuged (20,800 *g*, 10 min, 4 °C) and the supernatant (SNT) was stored. These steps were repeated for two times with 200 μl acetonitrile/methanol/water (2/2/1, v/v/v) skipping the heating. The SNTs of the three extraction cycles were pooled and stored for at least 2 h at −20 °C. After centrifuging the samples (20,800 *g*, 10 min, 4 °C) the SNT fluid was dried at 40 °C under a gentle nitrogen stream. The residual pellet was resolved in 150 μL water.

cNMP analysis of samples from bacterial culture medium (CM) was carried out by treating 50 μL CM with 200 μL of a mixture of acetonitrile/water (1/1, v/v). Then samples were heated for 15 min at 95 °C. After cooling down and storing for at least 2 h at −20 °C, samples were centrifuged (20,800 *g*, 10 min, 4 °C) and the supernatant fluid was dried at 40 °C under a gentle nitrogen stream. The residual pellet was resolved in 150 μL water containing 50 ng/mL of the internal standard (tenofovir).

### 2.4 Targeted HPLC/MS-MS measurement of cNMPs

cNMP detection and quantitation was performed via HPLC-MS/MS using a QTrap5500 triple quadrupole mass spectrometer (Sciex, Framingham, MA, United States) as described before (Beste et al., 2012; Bähre et al., 2014).

### 2.5 Non-targeted metabolomic analyses and determination of c-di-GMP concentration

For the metabolomic studies, a single colony was inoculated into 10 ml of fresh LB medium, supplemented with or without cUMP, and was incubated with aeration and vigorous shaking at 37 °C and 180 rpm for 20 h. A total of 5 ml of the culture were removed, centrifuged for 20 min, 2,500 *g*, 4 °C and resuspended in 500 μl fresh LB medium. The step was repeated once, and the pellet was extracted with ice cold acetonitrile/methanol/water (2/2/1; v/v/v) as described in further detail in a prior publication (Spangler et al., 2010). The extraction solvent was evaporated at 40 °C under a gentle nitrogen stream. The samples were dissolved in 100 μl water or methanol, centrifuged 10 min, 20,800 *g*, 4 °C, and 10 μl of each sample were combined to a quality control.

For determination of c-di-GMP concentration, 20 μl of the aqueous samples were saved to combine with 20 μl of internal standard and 40 μl water. Samples were analyzed with API 4000 LC-MS/MS (Sciex, Framingham, MA, United States) (Spangler et al., 2010).

The remaining volume of the samples was transferred into glass vials (Th. Geyer, Renningen, Germany) and analyzed in positive and negative ion spray mode with the VION IMS QTof mass spectrometer and coupled liquid chromatography system ACQUITY (Waters, New Castle, US-DE) with Eclipse XDB-C18 column (Agilent Santa Clara, United States). For separation, we used a reversed phase chromatography with linear increase of organic solvent from minute 3 to 17.2. For further analysis, the dataset was exported to ProgenesisQI (Waters) for peak picking, deconvolution, and identification of metabolites. Samples raw abundances were normalized on the amount of protein obtained from BCA Assay (Thermo Fischer, Waltham, United States) and statistically tested in MetaboAnalyst (Vers. 5.0, metaboanalyst.ca). A principal component analysis (PCA) was used to summarize the whole dataset. To verify the two identified metabolites, we compared the samples to HHQ and PQS obtained from Sigma Aldrich regarding retention time, mass-to-charge relation, collision cross section score (CCS), drift time, and fragmentation spectrum.

### 2.6 Bacterial growth curves

Prior to each experiment, a colony of PA14 was freshly inoculated from a VB-agar plate into 10 ml of LB medium prewarmed to 37 °C. The liquid culture was incubated with aeration and vigorous shaking at 37 °C and 180 rpm for 16 h. On the following day, the culture was diluted to an OD600 of 0.05 as determined with Ultrospec10 spectrometer (Amersham Biosciences, Freiburg, DE) and incubated with aeration and vigorous shaking at 37 °C and 180 rpm for another 6 h. For the measurements, this suspension was diluted to OD = 0.05 again, and 180 μL of the resulting suspension were transferred into each well of a 96 well plate (Sarstedt, Nümbrecht, DE). Cyclic nucleotides, vehicle or medium control were added to the wells in a volume of 20 μl. The microplates were incubated at 37 °C under continuous shaking in a Synergy 4 microplate reader (BioTek Instruments, Winooski, US-VT), and the absorption of the bacterial suspension was measured every 5 min at λ = 600 nm. In order to prevent edge or rim effects, the outermost wells of the plate had been filled with sterile PBS. The measurements were performed as technical triplicates with *n* = 5 independent replicates in total.

### 2.7 Determination of minimum inhibitory concentration (MIC)

Preparation of bacterial suspensions was performed analogous to the procedure mentioned above for bacterial growth curves. A total of 50 μL of the bacterial suspension (OD = 0.05) were pipetted into each well of a 96 well plate. A total of 100 μL of medium served as sterile control. Cyclic nucleotides or controls solved in medium were added in 10 μl volume. The respective antibacterial drug solved in medium was added in 40 μl to yield a total volume of 100 μl per well. After incubation on a plate shaker for 1 min, the plate was incubated at 37 °C for 16 h. After that, the absorption of the bacterial suspensions was measured at λ = 600 nm. We took the full concentration range of the drug to fit a 5-parameter logistic regression with shared top and bottom values to compare the calculated EC50 values. The 95% confidence intervals served as a threshold to determine statistically significant differences. In order to prevent edge or rim effects, the outermost wells of the plate had been filled with sterile PBS. The measurements were performed as technical duplicates with *n* = 5 independent replicates in total.

### 2.8 Assessment of bacterial biofilms

The assessment of bacterial biofilms has been done as described by Van Duuren et al. (2017). Briefly, the bacterial suspensions were prepared analogous to the procedure for bacterial growth curves as mentioned above. Experiments were performed with the xCELLigence^®^ measurement system on 16 well E-plates (both Agilent, Santa Clara, United States). For calibration, 50 μl of LB medium was pipetted into each well. The E-plate was placed in the incubator and equilibrated for 15 min before measuring the impedance once. In the meantime, the bacterial culture (in LB) was diluted to OD 0.13. The E-plate was removed and 100 μl of cNMPs, L-arginine, or medium control were transferred to each well before adding 150 μl of the bacterial suspension. The E-plate was placed back in the incubator. The impedance was measured every 15 min for at least 72 h. The measurements were performed in technical duplicates with *n* = 3 independent replicates in total.

### 2.9 Statistics

Statistical analyses were performed using GraphPad Prism, version 6 (Statcon). When two groups were compared, a two tailed *t*-test was performed. However, in the biofilm experiment, two tailed ratio paired *t*-tests with Bonferroni-Holm correction were used to compare the absolute slopes. For time to peak analysis and analyses comparing more than two groups One-Way- or Two-Way-ANOVAs (in case of two variables, e.g., time and substance) was performed. The assessment of statistical significance regarding the difference in growth kinetics was performed using non-linear regression (least squares fit) on the respective curves based on a five-parameter logistic function. It was then tested between two such datasets, if they are better described by a common regression using the same parameter (IC50/EC50/maximum; null hypothesis) or by regression functions using different parameters (alternative hypothesis). To test for significance, an extra sum-of-squares F test (α = 0.0001) was used. These analyses were performed using GraphPad Prism, version 10 (Statcon).

## 3 Results

**FIGURE 1:**
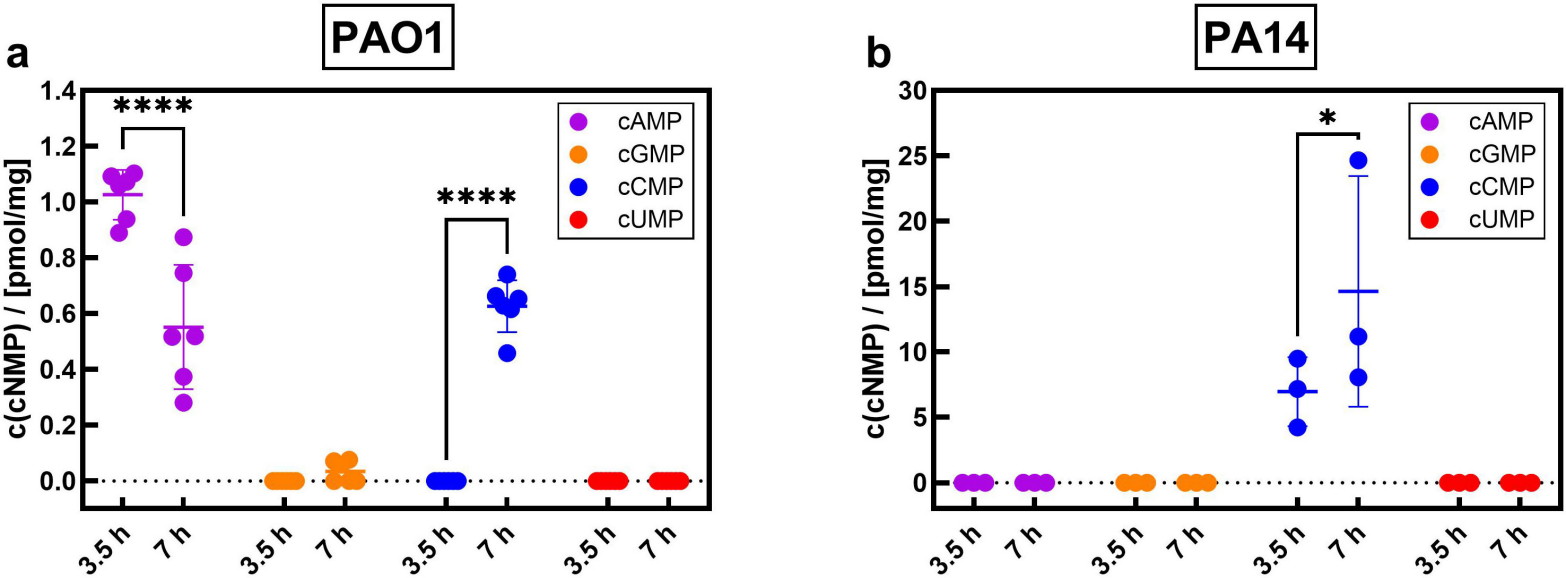
Measurement of endogenous cNMP synthesis in *P. aeruginosa* strains PAO1 **(a)** and PA14 **(b)** growing in Luria-Bertani (LB) medium in exponential growth phase (3.5 h) and stationary phase (7 h). At the indicated time points, bacteria were pelleted, extracted and prepared for HPLC-MS/MS based measurement of cyclic nucleotides. Shown are the means of six **(a)** or three **(b)** independent experiments with SD and the corresponding individual data points. *p < 0.05, **** *p* < 0.0001 with Two-Way ANOVA and *post hoc* Šídák correction.

When growing in a nutrient-rich medium such as LB medium, the intracellular concentrations of cGMP and cUMP do not differ between the logarithmic growth phase (3.5 h after inoculation) and the stationary phase (7 h after inoculation). In contrast, cAMP concentration decreased in the plateau phase in the PAO1 strain (7 h after inoculation), as compared to the concentrations determined in the exponential growth phase at 3.5 h after inoculation (Figure 1a). In PA14, we were not able to detect cAMP at all (Figure 1b). Interestingly, cCMP concentrations are significantly increased in both PAO1 and PA14 in the plateau phase as compared to the exponential growth phase (Figures 1a, b). In Vogel-Bonner (VB) minimum salt medium the growth phase-dependent increase of cCMP is completely lost, whereas the decrease of cAMP concentration can still be detected (Supplementary Figure 1).

**FIGURE 2:**
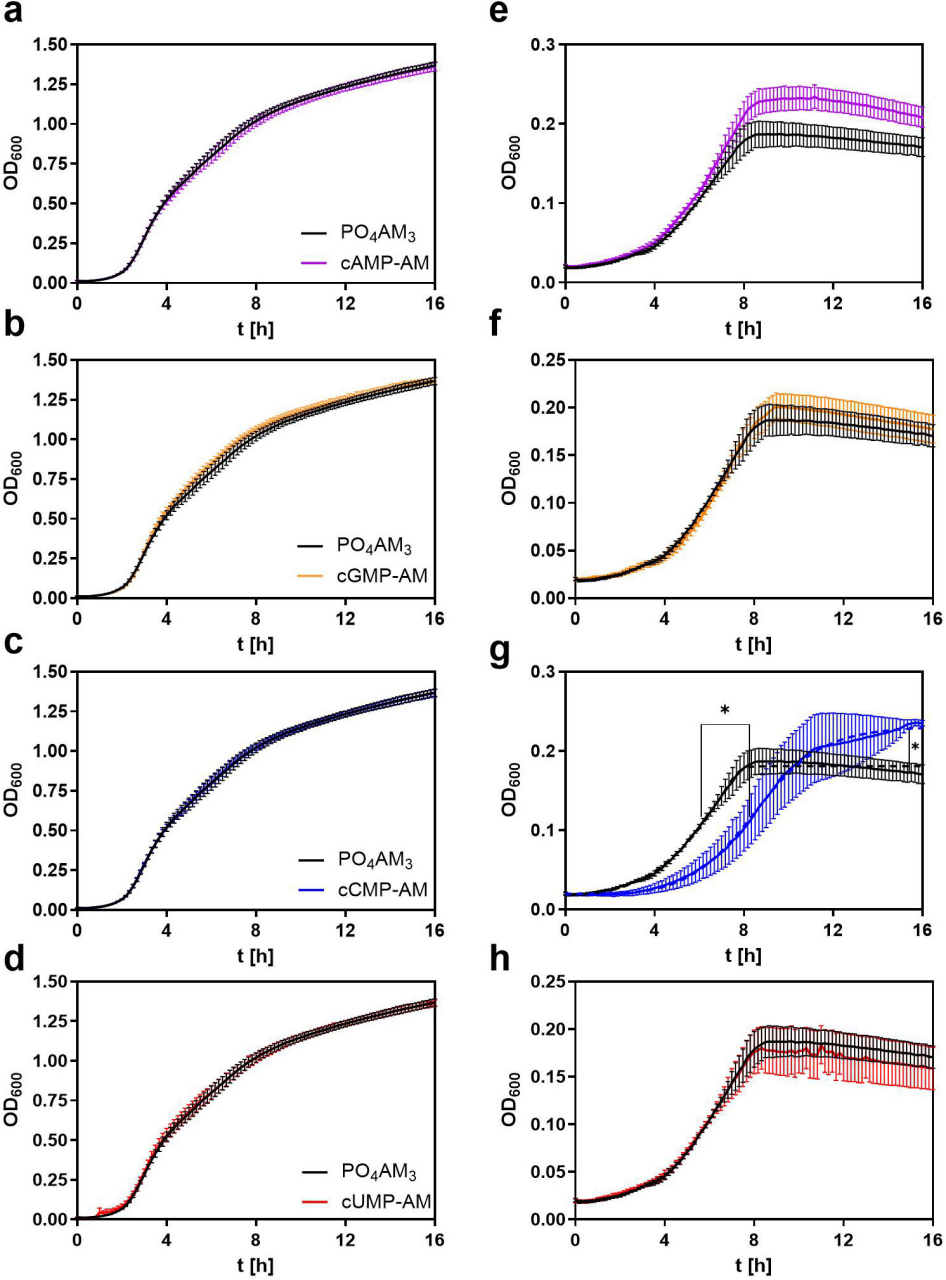
Growth curves of *P. aeruginosa* strain PA14 in Luria-Bertani (LB) **(a–d)** and Vogel-Bonner (VB) medium **(e–h)** with added cNMP-AMs in 100 μM. PO_4_AM_3_ was used in 33 μM as a control. The growth is determined by measuring the absorption at l = 600 nm every 10 min. Bacterial inocula were set to OD = 0.05 and incubated for 16 h in a 96 well plate. Here the canonical cAMP-AM **(a,e)** cGMP-AM **(b,f)** and non-canonical cNMP-AMs cCMP-AM **(c,g)** and cUMP-AM **(d,h)** were used. All samples contained the same amount of DMSO. Shown are the means ± S.E.M. of five independent experiments. *p < 0.0001 compared to control group with best-fit analysis of non-linear, five parameter logistic regression of growth curves and extra sum-of-squares F test.

In our laboratory the PA14 strain proved to be better suited for growth and biofilm experiments. In screening experiments, analyses of the PAO1 strain were less reproducible in terms of growth assay precision, and no cNMP-induced alteration of biofilm formation was observed. Indeed, compared to PAO1, PA14 has been described before as forming more adaptive and adaptive biofilms (Lee et al., 2020). Thus, we focused on this strain in all subsequent experiments. In LB growth medium, the PA14 growth kinetics have not been affected by any membrane-permeable cNMP analog (3’,5’-cNMP–AM) used in a screening concentration of 100 μM (Figures 2a–d, left panels). The PO_4_AM_3_ control showed a maximum growth rate at 3.08 ± 0.20 h. None of the growth kinetics determined in presence of cNMP-AM differed significantly from medium control in LB growth medium (Figures 2a–d, left panels). As a control, growth curves have been determined in presence of native cNMPs in a concentration of 100 μM. None of the native 3’,5’-cNMPs showed an effect here (Supplementary Figures 2a–d).

In VB medium, the maximum OD_600_ reached by the bacterial cultures was about 5-fold lower than in LB Medium and the maximum growth rate was reached at 6.08 ± 0.86 h (Figures 2e–h, right panels). In presence of cAMP-AM there was a slight overshoot of the growth curve as compared to the control curve, but no alteration of overall kinetics could be detected. In contrast, exposure to 100 μM cCMP-AM led to a delay in bacterial growth and shifted the maximum growth to 10.25 ± 2.58 h (Figure 2g). Neither cGMP-AM nor cUMP-AM had any effect on the growth in VB medium (Figures 2f, h, respectively). Like in LB medium, none of the native 3’,5’-cNMPs tested altered the growth kinetics of PA14 bacteria (Supplementary Figures 2e–h).

**FIGURE 3:**
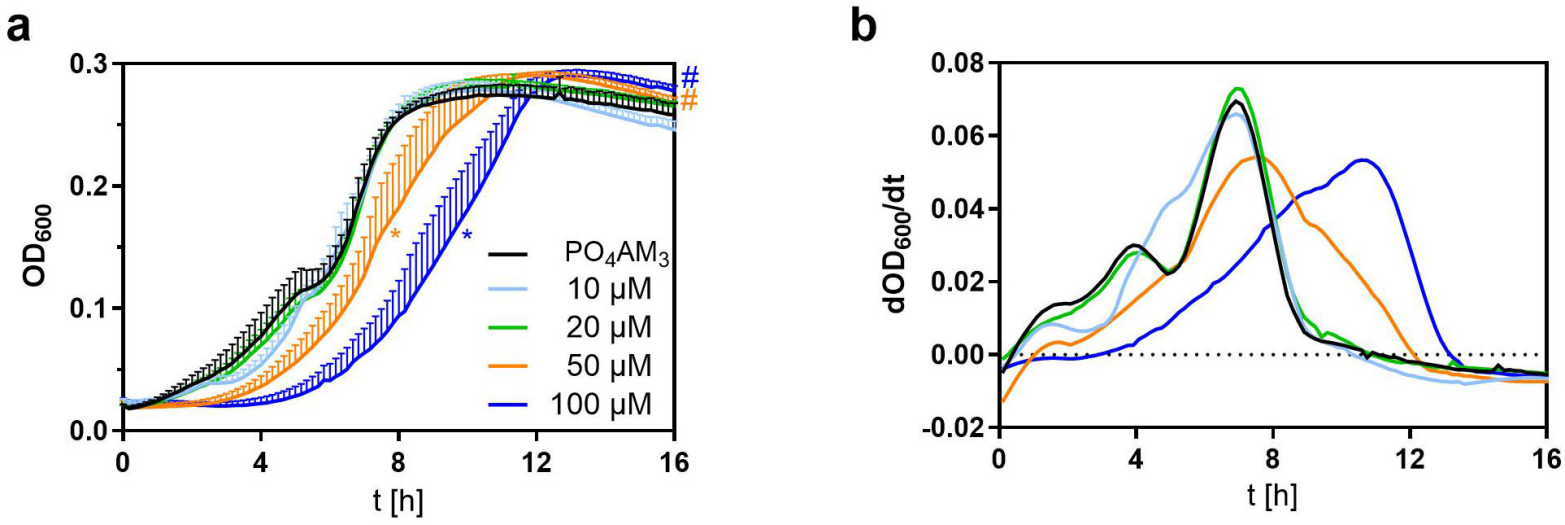
Inhibition of growth in Vogel-Bonner (VB) Medium with added 3’,5’-cyclic cytidine monophosphate (cCMP)-AM (10–100 μM). PO_4_AM_3_ in 33 μM was used as a control. **(a)** Shows the growth of *P. aeruginosa* by measuring the absorption at 600 nm every 10 min. Bacteria were set to OD = 0.05 and incubated for 16 h in a 96 well plate. **(b)** Shows the first derivative of the growth curves. On average, maximum growth peaks of 50 and 100 μM cCMP-AM are delayed by about 1.30 h (50 μM) and 3.14 h (100 μM) in comparison to PO_4_AM_3_. All samples contained the same amount of DMSO. Shown are the means of five independent experiments. *p < 0.0001 (EC 50), #*p* < 0.0001 (plateau height) compared to control group with best-fit analysis of non-linear, five parameter logistic regression of growth curves and extra sum-of-squares F test. The color of the asterisk/hashtags marks the respective treatment.

Since a retardation of bacterial growth by cCMP-AM was detectable in VB medium but not in LB medium, we chose to further investigate a dose dependency in VB medium, only. The observed effect of cCMP-AM on bacterial growth is concentration dependent: In presence of low concentrations (10, 20 μM) of cCMP-AM no difference against the PO_4_AM_3_ control is visible in the growth curves. At 50 μM cCMP-AM, a significant delay in growth could be detected whereas at 100 μM cCMP-AM an even more pronounced, significant shift of the growth curve can be observed (Figure 3a). As observed before in the screening experiments (Figure 2g), also the height of the final plateau was significantly increased beginning from 50 μM cCMP-AM (Figure 3a). Correspondingly, the first derivative curves of bacterial growth show a peak delay of about 1.3 h at 50 μM cCMP-AM and 3.14 h at 100 μM cCMP-AM (Figure 3b).

**FIGURE 4:**
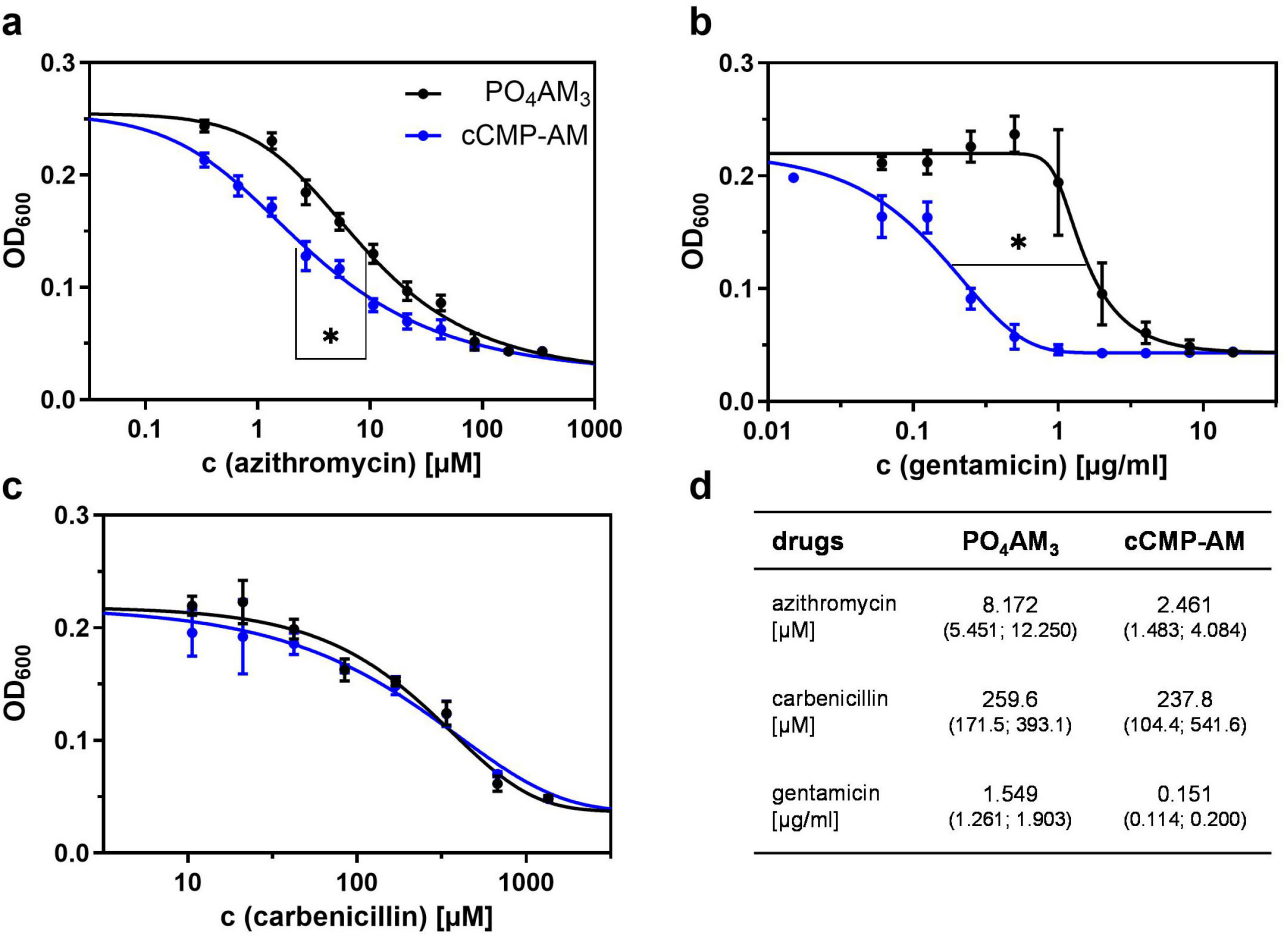
Sensitization against antibacterial drugs in Vogel-Bonner (VB) Medium. Bacteria were set to OD = 0.05 and incubated for 16 h in a 96 well plate. Final concentrations of 100 μM 3’,5’-cyclic cytidine monophosphate (cCMP)-AM and 33 μM PO_4_AM_3_ (control) were used. Antibacterial drug concentrations ranging from **(a)** 0.17–341.8 μM (azithromycin), **(b)** 0.016–16 μg/ml (gentamicin), and **(c)** 10.57–1353.06 μM (carbenicillin) in 2-fold steps. Shown are the means ± SD from three independent experiments. A five-parameter logistic regression was used to fit curves with shared global top and bottom parameters. Table **(d)** shows the calculated IC_50_ values with the 95% confidence intervals in brackets underneath. *p < 0.0001 compared to control group with best-fit analysis of non-linear, five parameter logistic regression of concentration effect curves and extra sum-of-squares F test.

To check if the effect of intracellular cCMP, as mimicked by membrane-permeable cCMP-AM, on growth might translate into a sensitization against common antibacterial drugs, we analyzed the impact of cCMP-AM combined with the antibacterial drugs azithromycin, carbenicillin, and gentamicin. In LB Medium, neither PO_4_AM_3_ control, nor cCMP, nor cCMP-AM altered antibacterial activity of the drugs tested (Supplementary Figure 3). In contrast, the efficacy of the antibacterial drugs was differentially affected by cCMP-AM when the analyses were performed in VB minimum salt medium (Figure 4). Bacterial growth inhibition by either azithromycin or gentamicin (Lacy et al., 1998; Firth and Prathapan, 2020) was increased significantly in presence of 100 μM cCMP-AM. The IC_50_ for azithromycin was reduced from 8.17 μM (PO_4_AM_3_ control) to 2.46 μM by cCMP-AM (Figures 4a, d). This effect is even more pronounced for gentamicin leading to a decimation of the IC_50_ from 1.55 to 0.151 μg/ml (Figures 4b, d). In contrast, antibacterial efficacy of carbenicillin was not altered by cCMP-AM at all (Figures 4c, d).

**FIGURE 5:**
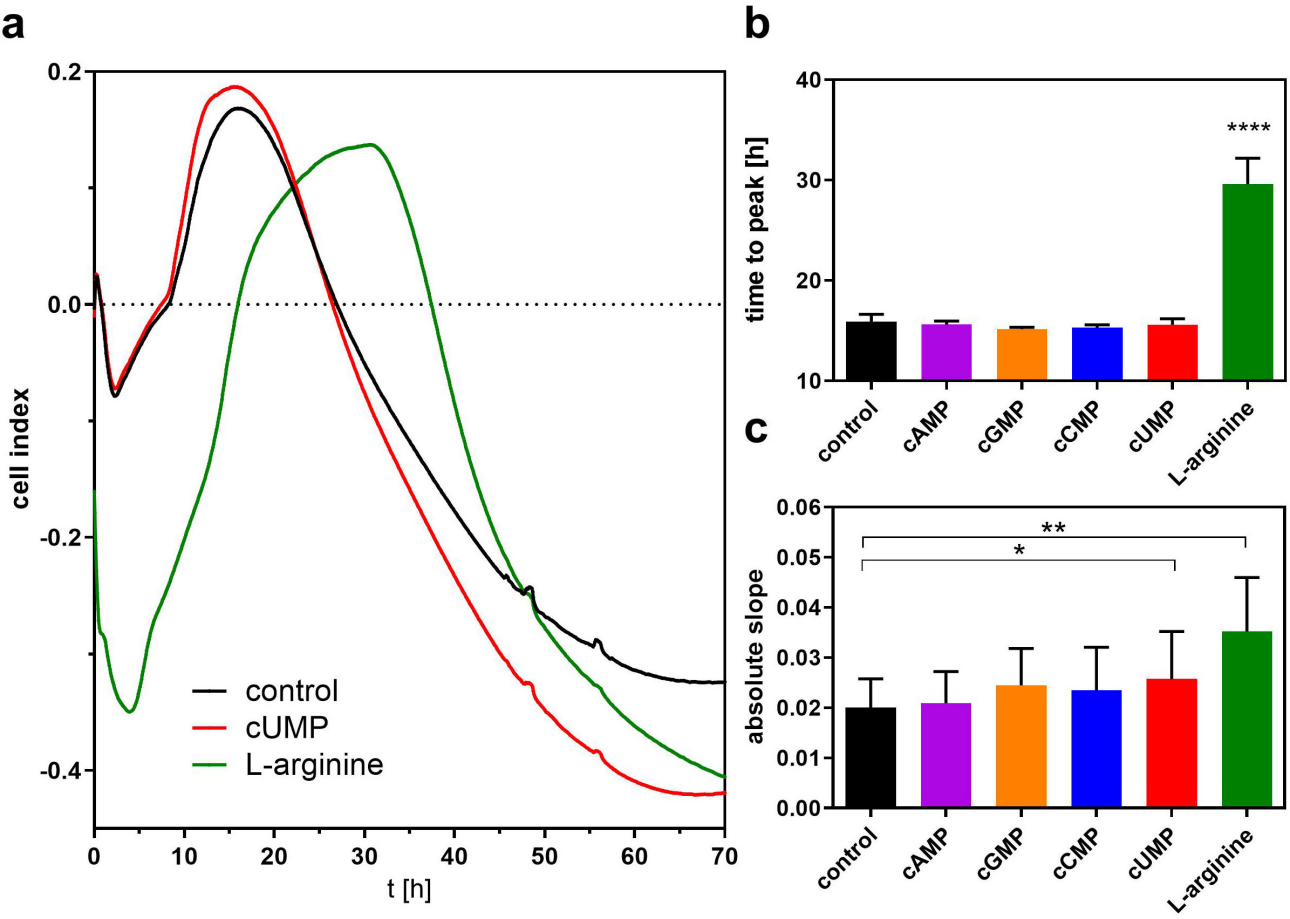
Assessment of bacterial biofilm formation. **(a)** Shows the pooled curve progression of 3’,5’-cyclic uridine monophosphate (cUMP), L-arginine and the control of three independent experiments detected by the xCelligence system. Bacteria were set to OD = 0.13 and grown for at least 70 h in Luria-Bertani (LB) Medium. Bacteria were stimulated with 100 μM cNMPs or 4.6 mM L-arginine. The control was performed without supplements. The time to peak is shown in **(b)**. The mean absolute linear slope of a 5 h interval is shown in **(c)**. For statistical analysis, a ratio paired two-tailed *t*-test was performed for absolute slope. For time to peak analysis, a one-way ANOVA was conducted. *p < 0.05; **p < 0.01; ****p < 0.0001.

In a second series of experiments, we screened for a possible effect of the cyclic nucleotides on biofilm formation as another key parameter of bacterial growth behavior in LB medium by measuring the change in bioimpedance in the xCELLigence^®^ real time analyzer. Biofilm formation appears after about 20 h when bacteria reach the stationary phase and is associated with the steepness of the cell index decrease in the xCELLigence^®^ system (Van Duuren et al., 2017). To validate our experiment, we used the amino acid L-arginine as control, which stimulates biofilm formation (Bernier et al., 2011; Rossi et al., 2022). For none of the native cNMPs did the global curve progression differ from the respective medium control in the first 20 h in contrast to L-arginine (Figure 5a) that led to a lag of about 13.69 ± 2.02 h compared to LB medium control to reach the cell index maximum (Figure 5b). Analyzing the slope of linear decrease correlating with biofilm formation, we detected a higher absolute slope in the cUMP-treated bacterial cultures than in the LB controls, but the highest slope in the L-arginine-treated samples (Figure 5c).

**FIGURE 6:**
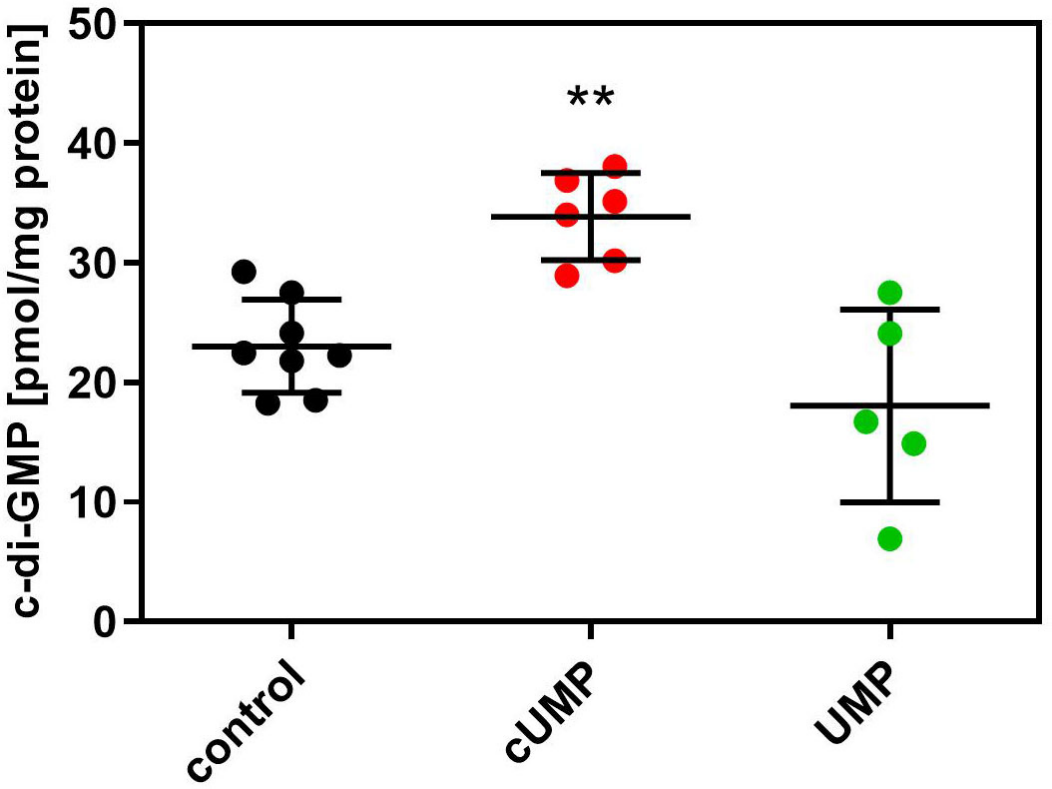
Intracellular (3’,5’)-cyclic guanosine monophosphate (c-di-GMP) concentration after an incubation for 20 h in Luria-Bertani (LB) Medium with or without 3’,5’-cyclic uridine monophosphate (cUMP) (10 μM). Measured c-di-GMP concentrations were normalized on mg protein of the extracted bacterial pellet determined in BCA assay. Shown are individual data points, their mean ± SD. Every data point represents one independent culture. *n* = 5–8; One-way ANOVA with Dunnett’s correction was performed to confirm statistical significant difference against the control. **p < 0.01.

Despite the calculatory significance of the cUMP effect, we wanted to validate the observed effect on biofilm formation, which seemed only very slight in the bioimpedance measurements. Therefore, we quantified intracellular c-di-GMP concentrations by HPLC-MS/MS (Figure 6). Since main changes in bioimpedance emerged after about 20 h, we determined c-di-GMP concentrations also at this time point and found a significant, 1.47-fold higher c-di-GMP concentration in cUMP-treated PA14 bacteria. The native, non-cyclic uridylyl monophosphate did not alter c-di-GMP concentrations significantly.

**FIGURE 7:**
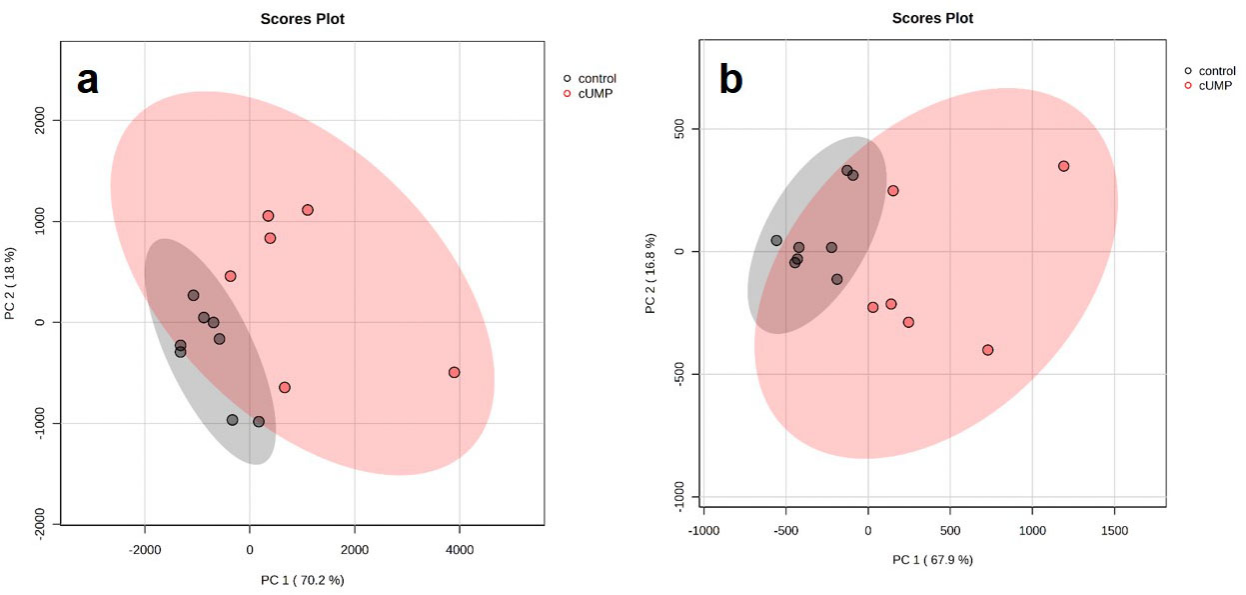
Principle component analysis from LC-QTOF data. A single colony was inoculated into 10 ml Luria-Bertani (LB) Medium and grown for 20 h stimulated with or without 3’,5’-cyclic uridine monophosphate (cUMP) (10 μM). Components were extracted and analyzed with MetaboAnalyst. Every data point represents one biological replicate. Colored regions show the estimated 95% confidence areas. **(a)** Samples analyzed in positive ionization mode. **(b)** Samples analyzed in negative ionization mode.

We subsequently prepared the same samples for untargeted mass spectrometric analyses in positive and negative ionization mode to uncover cUMP-induced changes in the bacterial metabolomes. Since UMP did not alter c-di-GMP concentrations, we focused on cUMP for further analyses. In a principal component analysis both the control samples and those treated with cUMP clustered in distinct groups, with higher overall variance in cUMP-stimulated samples. No cUMP-treated samples ranged inside the control’s 95% confidence area, indicating that at least some metabolomic pathways are regulated by cUMP (Figure 7).

**FIGURE 8:**
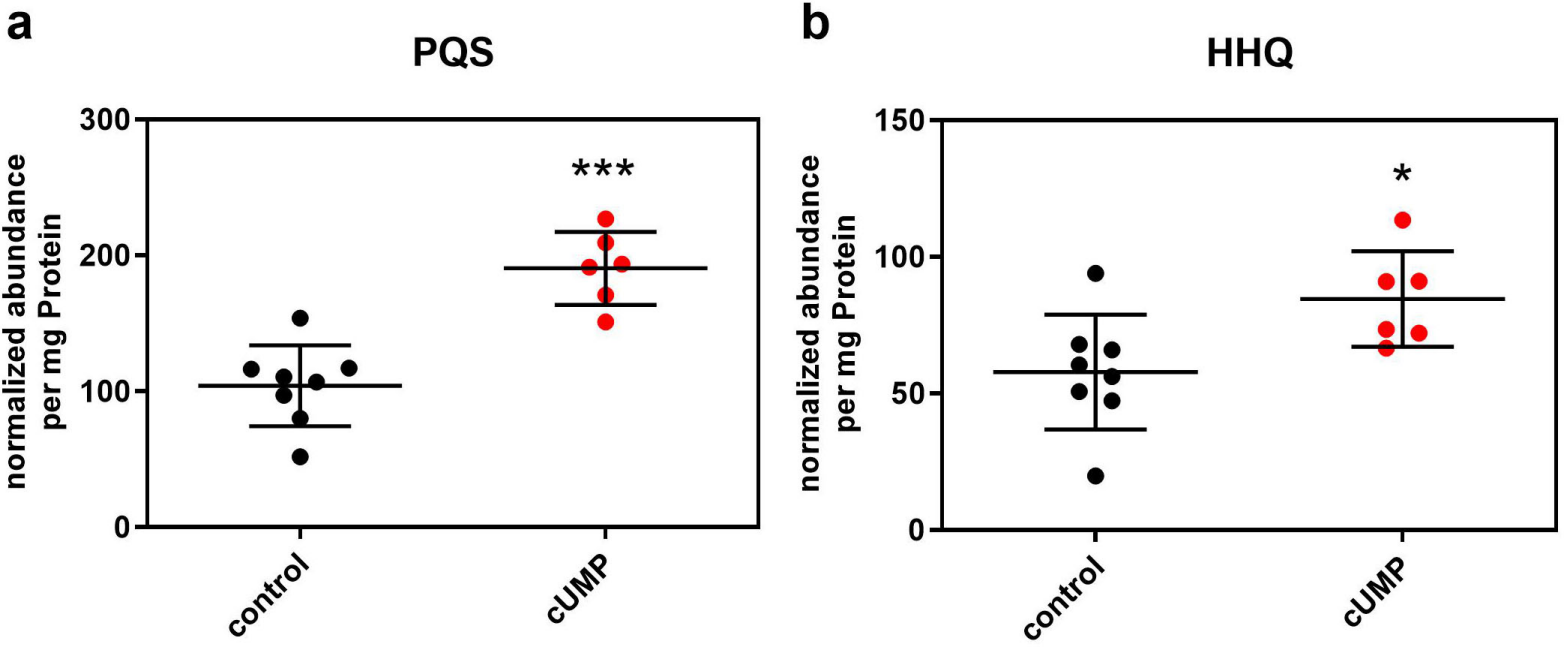
Detection of pseudomonas quinolone signal (PQS, panel **a**) and its precursor molecule 2-heptyl-4-hydroxyquinolone (HHQ, panel **b**) in LC-QTOF after 20 h of incubation in Luria-Bertani (LB) medium with or without 3’,5’-cyclic uridine monophosphate (cUMP) (10 μM). Raw abundance was normalized on the amount of protein of the extracted pellet determined in BCA-Assay. Shown are the individual replicates and their means ± SD. *n* = 8/6. Unpaired two-tailed *t*-test was performed to confirm statistical significance. *p < 0.05; ***p < 0.001.

Focusing on metabolites that influence biofilm formation, we could identify PQS and its direct precursor HHQ as two biofilm-regulating molecules from the pqs system in our samples by mass to charge relation, fragmentation, and verification with a HHQ- and PQS-containing standard mix. We then compared the raw abundances of these known autoinducers normalized on the amount of protein. cUMP-treated bacterial cultures showed significantly higher normalized abundances in HHQ and PQS with a mean fold change of 1.46 for HHQ and 1.83 for PQS (Figure 8).

The Supplementary Tables 1, 2 contains information on other compounds whose abundances were altered cUMP-dependently in our untargeted mass spectrometric analyses.

## 4 Discussion

Ever since the discovery of the anti-phage bacterial defense system Pycsar, cyclic CMP and UMP are moving more into focus of bacterial signal transduction research (Seifert and Schirmer, 2022). Our hypothesis was that cCMP and cUMP impact bacterial functions even when not affected by phage infections. Since the components of the Pycsar system are not present in the published genomes of the bacterial strains used in this study (Chen et al., 2023; Mukherjee et al., 2023), PA14 (and PAO1) are suitable models for these Pycsar-independent effects. In the present study, we could demonstrate that cCMP can be found endogenously in *P. aeruginosa* in the early stationary phase when growing in nutrient rich medium, whereas cUMP could not be detected under any condition in our analyses (Table 1). Production of cCMP when nutritive resource become limited after prior growth may lead to adaptation of the bacterial community to the scarce resources by restricting growth of bacteria or by initiating biofilm formation. Indeed, when applied to bacteria as membrane-permeable analog, cCMP led to a growth lag in *P. aeruginosa* in nutrient scarce VB-medium, whilst showing no effect on bacterial growth when applied to bacteria growing in nutrient rich medium (Table 1). This signaling may be analogous to the known role of cAMP in conjunction with CRP in carbon catabolite repression (cc) in *E. coli* (Jackson et al., 2002; Nanchen et al., 2008). In *P. aeruginosa*, the cAMP-CRP-complex is not a key player of catabolite repression. Instead, carbon source utilization is regulated in *P. aeruginosa* post-transcriptionally by the catabolite repression control (crc) protein, which is known to act as corepressor at target mRNA. Direct or indirect interaction of cCMP with the crc protein machinery might therefore be a possible mechanism underlying the growth inhibitory effect (Linares et al., 2010; Sonnleitner and Blä, 2014). Alternatively, cCMP may bind to and alter the conformation of a riboswitch, which is a known mechanism of transcriptional regulation after sensing c-di-GMP in *P. aeruginosa* (Sudarsan et al., 2008; Solano et al., 2009).

**TABLE 1:** Overview of the observed effects of cyclic pyrimidine nucleotides on *P. aeruginosa*.

Observation	cCMP	cCMP-AM	cUMP	cUMP-AM
Endogenous production	Yes	No	No	No
Effect on growth in nutrient-rich medium	Ø	Ø	Ø	Ø
Effect on growth in nutrient-poor medium	Ø	↓	Ø	Ø
Sensitization against azithromycin and gentamicin	No	Yes	No	No
Effect on biofilm formation	Ø	Ø	↑	Ø
Effect on c-di-GMP concentration	Ø	Ø	↑	Ø
Effect on PQS/HHQ concentration	Ø	Ø	↑	Ø

Ø, Not affected; ↑, Enhanced; ↓, Reduced.

The inhibitory effect of cCMP-AM on bacterial growth also translated into a sensitizing effect against certain antibacterial drugs when bacteria grow under nutrient scarce conditions (Table 1). While the IC_50_ of the β-lactam carbenicillin with regard to bacterial growth was not altered in presence of cCMP-AM, the aminoglycoside gentamicin and the macrolide azithromycin were more efficient in presence of the cyclic nucleotide. Carbenicillin is a bactericidal drug acting by inhibiting bacterial cell wall synthesis but is mainly used for *in vitro* studies. To test, if bacteria can be sensitized against β-lactams, antibacterial drugs that are used to treat *P. aeruginosa* infections in the clinics [e.g., cefepim (Britt et al., 2018)] should be tested in further studies. Both gentamicin and azithromycin act by inhibiting bacterial protein synthesis. Whilst gentamicin is known as bactericidal drug active against *P. aeruginosa* (Phe et al., 2019), azithromycin acts bacteriostatic and is indeed more suitable in *P. aeruginosa* infections, presumably because of its interference with quorum sensing (Hoffmann et al., 2007). The nucleotide structure of cCMP might hint toward a possible mechanism of the observed sensitization. Since the protein translation machinery is dependent on nucleotide-protein interactions, we hypothesize that cCMP might act as positive allosteric modulator at the ribosomal unit, thus enhancing binding of other bacterial drugs. This type of synergistic action is already known from the streptogramins with dalfopristin enhancing the binding of quinupristin at the 50S unit of the ribosome (Beyer and Pepper, 1998). An indirect action via the crc machinery or riboswitch binding, as discussed before, is another possible mechanism of action (Sudarsan et al., 2008; Solano et al., 2009; Linares et al., 2010; Sonnleitner and Blä, 2014). However, cCMP might represent a possible new lead structure for development for antibacterial drugs or additional drugs to boost established antibacterial substances in *P. aeruginosa* infections. Therefore, it is necessary to screen other *P. aeruginosa* strains including clinical isolates to verify the susceptibility regarding cCMP-AM.

The inhibitory effect on bacterial growth was concentration-dependent and vanished when native cCMP was used indicating that there is no uptake system for cCMP in *P. aeruginosa* or that there are potent efflux mechanisms or degrading enzymes present. Thus, cCMP must be provided by an intracellular generator to show the mentioned effect. A known specific generator of cCMP is the bacterial nucleotidyl cyclase PycC of *E. coli*, but the corresponding PycC of *P. aeruginosa* has been shown to produce cUMP only (Tal et al., 2021). The effector protein ExoY of *P. aeruginosa* has been shown to produce low concentrations of cCMP, but activity of the enzyme requires binding to host cell actin (Belyy et al., 2016). Consequently, the nucleotidyl cyclase responsible for the detected rise of cCMP after entering the stationary phase remains elusive.

It is known that the nutritional surrounding and growth conditions have influence on gene expression and metabolism in *P. aeruginosa* (Rossi et al., 2022; Dubern et al., 2023). Thus, it is not surprising that the cCMP-AM-dependent growth inhibition is rather “selective” for nutrient deficient VB medium in our experimental conditions. Further studies with titratable intracellular cCMP concentrations engaging inducible cCMP generators, using other membrane permanent cCMP analogs, or applying other growth medium compositions should also be tested to analyze the effects of metabolic state on the cCMP effect observed here.

Analyzing bacterial growth and biofilm formation by bioimpedance measurements, we were surprised to see no changes in bioimpedance kinetics in presence of any of the cNMPs or cNMP-AMs. But cUMP showed a stimulatory effect on biofilm formation as assessed by the surrogate parameter of bioimpedance decrease velocity after reaching a peak. The effect was significant but subtle, so we sought further validation of this finding with another experimental approach. Indeed, we were able to detect elevated intracellular concentrations of cyclic di-GMP in response to native cUMP. Since c-di-GMP is a known regulator of biofilm formation in *P. aeruginosa* (7–9), this finding corroborated the conclusion drawn from the bioimpedance measurement (Table 1).

In previous studies we detected huge amounts of cUMP after infection of mammalian cells or mice lungs with *P. aeruginosa* harboring the cUMP-producing enzyme ExoY. The effector protein is a well-known nucleotidyl cyclase with preference for GTP and UTP as substrates depending on the infected mammalian cell line (Beckert et al., 2014). During infection ExoY is injected via the bacterial type III secretion system into the host cell, and, after binding host actin, produces significant amounts of cUMP. Since cUMP can subsequently be transported out of mammalian cells by the multidrug resistance proteins MRP4 and MRP5 (Seifert, 2015) or after lytic cell death, it may serve as signal for a successful infection and improve bacterial residence by increasing biofilm formation. Indeed, the high concentrations of cUMP found in the urine of mice infected with *P. aeruginosa* expressing ExoY can be regarded as result of this cellular release during infection.

To test if the cUMP-induced signaling may affect other metabolic pathways in the bacteria, we performed a mass spectrometry-based metabolomic screening. Within the several identified compounds regulated by cUMP, we were able to detect and validate the Pseudomonas Quinolone Signal (PQS) and its pathway precursor HHQ (Table 1). The main AHLs from las and rhl system could not be detected. Thus, cUMP might affect biofilm formation not only by influencing c-di-GMP signaling, but by acting via the quorum sensing pathway, too.

Administration of quorum sensing inhibitors is an attractive therapeutic strategy, since development of resistance against these inhibitors is low (Pang et al., 2019). Thus, uncovering the interaction mechanism between cUMP and the *pqs* pathway is of high interest. By targeting synthesizing enzymes or treatment with precursor molecule analogs, the cUMP-dependent increase of PQS concentration could be diminished (Reuter et al., 2016).

Our experiments prove that *P. aeruginosa* is able to detect cUMP in the environment and consequently regulate its metabolome and ability to form biofilm. The findings were only detectable with cUMP but not with the membrane permeable cUMP-AM. Thus, we presume that cUMP serves first messenger roles binding to an extracellular receptor. 3’,5’-Cyclic nucleotides are quite stable in the absence of their respective PDEs (Reinecke et al., 2011). In the context of the Pycsar system, it has been shown, that cUMP or cCMP are stable for up to 20 h in mixtures of these nucleotides with bacterial lysates, indicating the absence of PDE activity in this context. However, if bacteria are infected with phages that subvert the Pycsar immunity by introducing enzymes with PDE activity, the cNMPs degrade rapidly (Tal et al., 2021; Hobbs et al., 2022). But, of course, there may be other mechanisms, such as transport or inducible enzymes with PDE activity, that have yet to be discovered. For example, an ekto-phosphodiesterase for cUMP may exist, which degrades cUMP to uridine. This could be transported through the cell membrane and trigger the observed reaction. That would explain why cUMP-AM did not show the same response as cUMP.

On the one hand, a possible detector of cUMP could be the GacS/GacA system part of the widespread two component regulatory system in bacteria (Zschiedrich et al., 2016). GacS has an extracellular domain with an unclear activation signal (Fadel et al., 2022). On the intracellular site is a histidine kinase which, in the activated state, phosphorylates the regulation protein GacA. The two small regulatory RNAs RsmY und RsmZ are subsequently increasingly expressed and activate QS by binding to the small RNA binding protein RsmA (Nadal Jimenez et al., 2012). In consequence pqs and the c-di-GMP systems are activated and lead to increased biofilm formation (Rasamiravaka et al., 2015). To prove our hypothesis, further experiments focusing on the GacS system are necessary to characterize the binding profile of the extracellular domain.

On the other hand, uridine imported into bacterial cells may be funneled into the uridine diphosphate (UDP) sugar metabolism. UDP sugars are used in *P. aeruginosa* as glycosyl donors for the synthesis of virulence factors and matrix polysaccharides such as the exopolysaccharide *Psl* (Priebe et al., 2004; Berbis et al., 2015; Dirr et al., 2024). Via this mechanism, extracellular cUMP could increase biofilm formation by simply filling the reservoir of nucleotide building blocks necessary for polysaccharide synthesis.

Our findings regarding the cUMP-dependent modulation of biofilm production by PA14 raise questions about a potential involvement of this cyclic pyrimidine nucleotide in different disease contexts. Biofilms observed in chronic *P. aeruginosa* infections in cystic fibrosis (CF) patients differ significantly from those found in patients with ventilator-associated pneumonias (VAP) (Fricks-Lima et al., 2011). In a murine model of acute lung infection with a *P. aeruginosa* strain that expresses supranormal amounts of the bacterial nucleotidyl cyclase ExoY, we have observed significant accumulation of cUMP in the lung tissue and a substantial spillover into the mice’s sera (Kloth et al., 2018; Munder et al., 2018). Due to its cUMP-dependent biofilm behavior observed in this study and its ability to adapt its surface attachment and biofilm synthesis to environmental conditions (Lee et al., 2020), PA14 is a promising candidate for further investigating cUMP effects in chronic models of *Pseudomonas* infections. Furthermore, clinical samples from CF or VAP patients could be valuable for analyzing cUMP concentrations and associating them with different disease conditions.

## Conclusion

**FIGURE 9:**
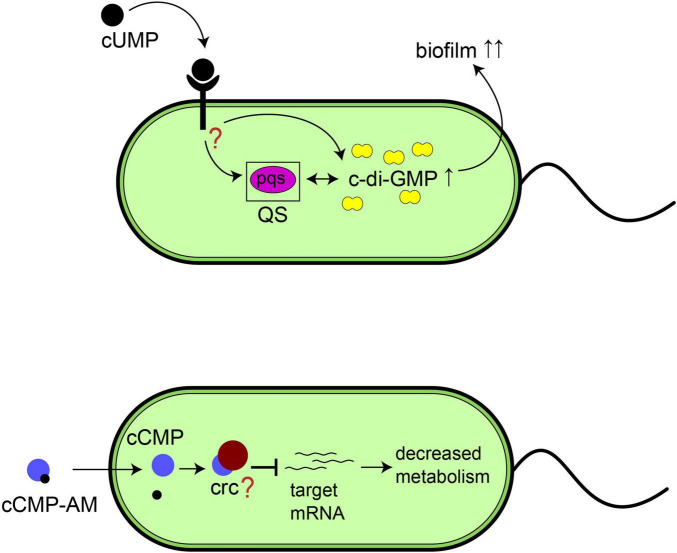
Proposed functions of cyclic pyrimidine nucleotides in *P. aeruginosa.* (Top) Schematic representation of the effect of extracellular 3’,5’-cyclic uridine monophosphate (cUMP) on biofilm formation via c-di-GMP or the quorum sensing system. (Bottom) Schematic representation of the possible effect of 3’,5’-cyclic cytidine 26 monophosphate (cCMP) on bacterial growth.

Our study suggests new biological functions of the cyclic pyrimidine nucleotides cCMP and cUMP in bacteria (Figure 9). Although only the corresponding nucleobase is changed, the pyrimidine nucleotides have different effects in *P. aeruginosa*. cCMP-AM as surrogate for intracellular cCMP decreased bacterial growth and reduced the minimum inhibitory concentrations (MIC) of azithromycin and gentamicin. Therefore, cCMP might be an interesting lead compound for development of drugs boosting the effectivity of antibacterial drugs.

In contrast to cCMP, native cUMP turned out to be an environmental inductor of the quorum sensing system pqs and intracellular c-di-GMP in our experiments. Based on our previous observations of the *P. aeruginosa* effector protein ExoY producing huge amounts of cUMP during acute infections, we consider cUMP as a signal for a successful infection consequently stimulating residence by e.g., increased biofilm formation.

## Data Availability

The raw data supporting the conclusions of this article will be made available by the authors, without undue reservation.
