# Supplementary material for: The effect of cCMP and cUMP on growth of Pseudomonas aeruginosa
Source: Front Microbiol. 2025 Sep 16;16:1675794. doi: 10.3389/fmicb.2025.1675794 (PMC12489823; doi:10.3389/fmicb.2025.1675794)
Supplement: Supplementary file 1 [file Table_1.docx]

Supplementary Table 1: Other compounds identified as significantly (p<0.05; ANOVA) changed upon exposure to cUMP in the untargeted mass spectrometric screening (positive ionization mode)

| **Compound** | **Compound ID** | **Adducts** | **Formula** | **Mass Error (ppm)** | **Isotope Similarity** | **Description** | **m/z** | **Charge** | **Retention time (min)** | **Anova (p)** | **Max Fold Change** |
| --- | --- | --- | --- | --- | --- | --- | --- | --- | --- | --- | --- |
| 1.08_313.1181m/z | CSID125 | 2M+H | C6H8N2O3 | 12,25 | 98,80 | Imidazol-4-one-5-propionic acid | 313,12 | 1 | 1,08 | 0,00000 | Infinity |
| 1.08_313.1181m/z | CSID2340020 | M+ACN+H | C15H13NO4 | -0,75 | 90,46 | p-Hydroxyketorolac | 313,12 | 1 | 1,08 | 0,00000 | Infinity |
| 1.08_313.1181m/z | CSID35013487 | M+H-2H2O | C12H20N4O8 | 11,02 | 91,45 | N~2~-[2-(Carboxymethyl)-2-hydroxy-3-methoxy-3-oxopropanoyl]arginine | 313,12 | 1 | 1,08 | 0,00000 | Infinity |
| 1.08_313.1181m/z | CSID404046 | 2M+H | C6H8N2O3 | 12,25 | 98,80 | Imidazolelactic acid | 313,12 | 1 | 1,08 | 0,00000 | Infinity |
| 1.08_313.1181m/z | CSID5960 | 2M+H | C11H8O | -13,46 | 95,56 | naphthal | 313,12 | 1 | 1,08 | 0,00000 | Infinity |
| 1.08_313.1181m/z | CSID5966 | 2M+H | C11H8O | -13,46 | 95,56 | QJ0190010 | 313,12 | 1 | 1,08 | 0,00000 | Infinity |
| 1.08_313.1181m/z | CSID74849619 | 2M+H | C6H8N2O3 | 12,25 | 98,80 | 3-(5-Hydroxy-1H-imidazol-4-yl)propanoic acid | 313,12 | 1 | 1,08 | 0,00000 | Infinity |
| 1.08_313.1181m/z | CSID8635 | 2M+H | C6H8N2O3 | 12,25 | 98,80 | pentoxyl | 313,12 | 1 | 1,08 | 0,00000 | Infinity |
| 1.08_313.1181m/z | CSID8643819 | M+ACN+H | C15H13NO4 | -0,75 | 90,46 | 1,6-Dihydroxy-3-methoxy-10-methyl-9(10H)-acridinone | 313,12 | 1 | 1,08 | 0,00000 | Infinity |
| 1.08_313.1181m/z | CSID9359907 | M+ACN+H | C15H13NO4 | -0,75 | 90,46 | 6,7-Dimethoxy-3-methyl-1H-carbazole-1,4(9H)-dione | 313,12 | 1 | 1,08 | 0,00000 | Infinity |
| 15.57_288.2515m/z | CSID4576432 | M+ACN+H | C14H30O3 | -7,59 | 90,19 | Hydroxycitronellal diethyl acetal | 288,25 | 1 | 15,57 | 0,00005 | 133,05 |
| 16.81_230.2119m/z | CSID12206 | M+NH4 | C13H24O2 | 2,34 | 95,10 | YQ2978400 | 230,21 | 1 | 16,81 | 0,00033 | 203,77 |
| 16.81_230.2119m/z | CSID14837 | M+NH4 | C13H24O2 | 2,34 | 95,10 | CYCLOTRIDECANOLIDE | 230,21 | 1 | 16,81 | 0,00033 | 203,77 |
| 16.81_230.2119m/z | CSID20143209 | M+NH4 | C13H24O2 | 2,34 | 95,10 | (2Z,6Z)-1,1-Diethoxy-2,6-nonadiene | 230,21 | 1 | 16,81 | 0,00033 | 203,77 |
| 16.81_230.2119m/z | CSID35013493 | M+NH4 | C13H24O2 | 2,34 | 95,10 | (4E)-3-Methyl-4-decen-1-yl acetate | 230,21 | 1 | 16,81 | 0,00033 | 203,77 |
| 16.81_230.2119m/z | CSID35014445 | M+NH4 | C13H24O2 | 2,34 | 95,10 | 3-Octanyl (2Z)-2-methyl-2-butenoate | 230,21 | 1 | 16,81 | 0,00033 | 203,77 |
| 16.81_230.2119m/z | CSID35014878 | M+NH4 | C13H24O2 | 2,34 | 95,10 | 4-(3-Hydroxybutyl)-3,3,5-trimethylcyclohexanone | 230,21 | 1 | 16,81 | 0,00033 | 203,77 |
| 16.81_230.2119m/z | CSID54994 | M+NH4 | C13H24O2 | 2,34 | 95,10 | 10-Undecen-1-yl acetate | 230,21 | 1 | 16,81 | 0,00033 | 203,77 |
| 16.81_230.2119m/z | CSID553318 | M+NH4 | C13H24O2 | 2,34 | 95,10 | Methyl dodec-2-enoate | 230,21 | 1 | 16,81 | 0,00033 | 203,77 |
| 16.81_230.2119m/z | CSID56046 | M+NH4 | C13H24O2 | 2,34 | 95,10 | CONIFER ACETATE | 230,21 | 1 | 16,81 | 0,00033 | 203,77 |
| 16.81_230.2119m/z | CSID58891 | M+NH4 | C13H24O2 | 2,34 | 95,10 | 2,6,10,10-Tetramethyl-1-oxaspiro[4.5]decan-6-ol | 230,21 | 1 | 16,81 | 0,00033 | 203,77 |
| 16.81_230.2119m/z | CSID7493 | M+NH4 | C13H24O2 | 2,34 | 95,10 | RHODINYL PROPIONATE | 230,21 | 1 | 16,81 | 0,00033 | 203,77 |
| 16.81_230.2119m/z | CSID79223 | M+ACN+H | C11H24O2 | 2,63 | 97,15 | 1,1-Dimethoxynonane | 230,21 | 1 | 16,81 | 0,00033 | 203,77 |
| 16.81_230.2119m/z | CSID8502 | M+NH4 | C13H24O2 | 2,34 | 95,10 | RH3487500 | 230,21 | 1 | 16,81 | 0,00033 | 203,77 |
| 12.24_234.2057m/z | CSID22928 | M+NH4 | C12H24O3 | -3,24 | 95,93 | 5-Hydroxydodecanoic acid | 234,21 | 1 | 12,24 | 0,00041 | 167,82 |
| 12.24_234.2057m/z | CSID4472229 | M+NH4 | C12H24O3 | -3,24 | 95,93 | (-)-3-Hydroxydodecanoic acid | 234,21 | 1 | 12,24 | 0,00041 | 167,82 |
| 12.24_234.2057m/z | CSID71366 | M+NH4 | C12H24O3 | -3,24 | 95,93 | 12-Hydroxylauric acid | 234,21 | 1 | 12,24 | 0,00041 | 167,82 |
| 12.24_234.2057m/z | CSID85026 | M+NH4 | C12H24O3 | -3,24 | 95,93 | MFCD00133279 | 234,21 | 1 | 12,24 | 0,00041 | 167,82 |
| 14.20_262.2372m/z | CSID11486376 | M+NH4 | C14H28O3 | -1,97 | 95,66 | 3-[(2-Isopropyl-5-methylcyclohexyl)oxy]-2-methyl-1,2-propanediol | 262,24 | 1 | 14,20 | 0,00133 | 28,34 |
| 14.20_262.2372m/z | CSID1508 | M+NH4 | C14H28O3 | -1,97 | 95,66 | 2-Hydroxymyristic acid | 262,24 | 1 | 14,20 | 0,00133 | 28,34 |
| 14.20_262.2372m/z | CSID15252 | M+NH4 | C14H28O3 | -1,97 | 95,66 | MFCD00059633 | 262,24 | 1 | 14,20 | 0,00133 | 28,34 |
| 14.20_262.2372m/z | CSID4450466 | M+NH4 | C14H28O3 | -1,97 | 95,66 | (3R)-3-hydroxymyristic acid | 262,24 | 1 | 14,20 | 0,00133 | 28,34 |
| 14.20_262.2372m/z | CSID500647 | M+NH4 | C14H28O3 | -1,97 | 95,66 | Ethyl 3-hydroxydodecanoate | 262,24 | 1 | 14,20 | 0,00133 | 28,34 |
| 15.34_202.1789m/z | CSID102576 | M+ACN+H | C9H20O2 | -7,58 | 95,12 | 1-ethoxy-1-pentoxyethane | 202,18 | 1 | 15,34 | 0,00319 | 6,29 |
| 15.34_202.1789m/z | CSID10771160 | M+NH4 | C11H20O2 | -6,59 | 93,11 | 10-Undecenoic acid | 202,18 | 1 | 15,34 | 0,00319 | 6,29 |
| 15.34_202.1789m/z | CSID148120 | M+NH4 | C11H20O2 | -6,59 | 93,11 | 3-Acetoxy-1-nonene | 202,18 | 1 | 15,34 | 0,00319 | 6,29 |
| 15.34_202.1789m/z | CSID151463 | M+NH4 | C11H20O2 | -6,59 | 93,11 | Prenyl caproate | 202,18 | 1 | 15,34 | 0,00319 | 6,29 |
| 15.34_202.1789m/z | CSID19046 | M+NH4 | C11H20O2 | -6,59 | 93,11 | RH0365000 | 202,18 | 1 | 15,34 | 0,00319 | 6,29 |
| 15.34_202.1789m/z | CSID20138479 | M+NH4 | C11H20O2 | -6,59 | 93,11 | PEARLATE | 202,18 | 1 | 15,34 | 0,00319 | 6,29 |
| 15.34_202.1789m/z | CSID251275 | M+NH4 | C11H20O2 | -6,59 | 93,11 | 4-Methyl-4-decanolide | 202,18 | 1 | 15,34 | 0,00319 | 6,29 |
| 15.34_202.1789m/z | CSID253449 | M+NH4 | C11H20O2 | -6,59 | 93,11 | MFCD00046351 | 202,18 | 1 | 15,34 | 0,00319 | 6,29 |
| 15.34_202.1789m/z | CSID30776879 | M+NH4 | C11H20O2 | -6,59 | 93,11 | (3Z)-3-Methyl-3-decenoic acid | 202,18 | 1 | 15,34 | 0,00319 | 6,29 |
| 15.34_202.1789m/z | CSID30776896 | M+NH4 | C11H20O2 | -6,59 | 93,11 | Ethyl (2Z)-2-nonenoate | 202,18 | 1 | 15,34 | 0,00319 | 6,29 |
| 15.34_202.1789m/z | CSID35013311 | M+NH4 | C11H20O2 | -6,59 | 93,11 | (4Z)-3-Methyl-4-decenoic acid | 202,18 | 1 | 15,34 | 0,00319 | 6,29 |
| 15.34_202.1789m/z | CSID4509339 | M+NH4 | C11H20O2 | -6,59 | 93,11 | (2E)-Hex-2-enyl 2-methylbutyrate | 202,18 | 1 | 15,34 | 0,00319 | 6,29 |
| 15.34_202.1789m/z | CSID4509340 | M+NH4 | C11H20O2 | -6,59 | 93,11 | trans-2-Hexenyl Isovalerate | 202,18 | 1 | 15,34 | 0,00319 | 6,29 |
| 15.34_202.1789m/z | CSID4509760 | M+NH4 | C11H20O2 | -6,59 | 93,11 | (2E)-2-Hexenyl pentanoate | 202,18 | 1 | 15,34 | 0,00319 | 6,29 |
| 15.34_202.1789m/z | CSID4515527 | M+NH4 | C11H20O2 | -6,59 | 93,11 | (2Z)-2-Pentenyl hexanoate | 202,18 | 1 | 15,34 | 0,00319 | 6,29 |
| 15.34_202.1789m/z | CSID4517162 | M+NH4 | C11H20O2 | -6,59 | 93,11 | MFCD00036530 | 202,18 | 1 | 15,34 | 0,00319 | 6,29 |
| 15.34_202.1789m/z | CSID4519169 | M+NH4 | C11H20O2 | -6,59 | 93,11 | (3Z)-Hex-3-en-1-yl isovalerate | 202,18 | 1 | 15,34 | 0,00319 | 6,29 |
| 15.34_202.1789m/z | CSID4519170 | M+NH4 | C11H20O2 | -6,59 | 93,11 | 3-Hexenyl pentanoate | 202,18 | 1 | 15,34 | 0,00319 | 6,29 |
| 15.34_202.1789m/z | CSID4519468 | M+NH4 | C11H20O2 | -6,59 | 93,11 | Methyl decenoate | 202,18 | 1 | 15,34 | 0,00319 | 6,29 |
| 15.34_202.1789m/z | CSID473227 | M+NH4 | C11H20O2 | -6,59 | 93,11 | (2-Hexylcyclopropyl)acetic acid | 202,18 | 1 | 15,34 | 0,00319 | 6,29 |
| 15.34_202.1789m/z | CSID4829022 | M+NH4 | C11H20O2 | -6,59 | 93,11 | 3-Hexenyl 2-methylbutyrate | 202,18 | 1 | 15,34 | 0,00319 | 6,29 |
| 15.34_202.1789m/z | CSID4941719 | M+NH4 | C11H20O2 | -6,59 | 93,11 | trans-3-Heptenyl isobutyrate | 202,18 | 1 | 15,34 | 0,00319 | 6,29 |
| 15.34_202.1789m/z | CSID55044 | M+NH4 | C11H20O2 | -6,59 | 93,11 | Rhodinyl formate | 202,18 | 1 | 15,34 | 0,00319 | 6,29 |
| 15.34_202.1789m/z | CSID55148 | M+NH4 | C11H20O2 | -6,59 | 93,11 | UQ1320000 | 202,18 | 1 | 15,34 | 0,00319 | 6,29 |
| 15.34_202.1789m/z | CSID55229 | M+NH4 | C11H20O2 | -6,59 | 93,11 | Methyl rhodinolate | 202,18 | 1 | 15,34 | 0,00319 | 6,29 |
| 15.34_202.1789m/z | CSID55333 | M+NH4 | C11H20O2 | -6,59 | 93,11 | 25ODI8S42W | 202,18 | 1 | 15,34 | 0,00319 | 6,29 |
| 15.34_202.1789m/z | CSID55376 | M+ACN+H | C9H20O2 | -7,58 | 95,12 | 1,1-Dimethoxyheptane | 202,18 | 1 | 15,34 | 0,00319 | 6,29 |
| 15.34_202.1789m/z | CSID55387 | M+NH4 | C11H20O2 | -6,59 | 93,11 | MFCD00001526 | 202,18 | 1 | 15,34 | 0,00319 | 6,29 |
| 15.34_202.1789m/z | CSID66412 | M+NH4 | C11H20O2 | -6,59 | 93,11 | Cyclohexyl valerate | 202,18 | 1 | 15,34 | 0,00319 | 6,29 |
| 15.34_202.1789m/z | CSID69650 | M+ACN+H | C9H20O2 | -7,58 | 95,12 | 1,1-Diethoxypentane | 202,18 | 1 | 15,34 | 0,00319 | 6,29 |
| 15.34_202.1789m/z | CSID7428 | M+NH4 | C11H20O2 | -6,59 | 93,11 | YQ2485000 | 202,18 | 1 | 15,34 | 0,00319 | 6,29 |
| 15.34_202.1789m/z | CSID7490 | M+NH4 | C11H20O2 | -6,59 | 93,11 | RH3480000 | 202,18 | 1 | 15,34 | 0,00319 | 6,29 |
| 15.34_202.1789m/z | CSID9216580 | M+NH4 | C11H20O2 | -6,59 | 93,11 | cis-5-Octenyl propionate | 202,18 | 1 | 15,34 | 0,00319 | 6,29 |
| 15.34_202.1789m/z | CSID96085 | M+NH4 | C11H20O2 | -6,59 | 93,11 | ÃŽÂ²-Methyl-ÃŽÂ³-decalactone | 202,18 | 1 | 15,34 | 0,00319 | 6,29 |
| 15.34_202.1789m/z | CSID98237 | M+NH4 | C11H20O2 | -6,59 | 93,11 | 2,4-Dimethyl-2-(4-methylpent-3-en-1-yl)-1,3-dioxolane | 202,18 | 1 | 15,34 | 0,00319 | 6,29 |
| 15.32_224.1597m/z | CSID101032 | 2M+NH4 | C4H9NO2 | -3,88 | 94,90 | 2-Aminoethyl acetate | 224,16 | 1 | 15,32 | 0,00342 | 7,10 |
| 15.32_224.1597m/z | CSID10469 | 2M+NH4 | C4H9NO2 | -3,88 | 94,90 | EK7713333 | 224,16 | 1 | 15,32 | 0,00342 | 7,10 |
| 15.32_224.1597m/z | CSID10530 | 2M+NH4 | C4H9NO2 | -3,88 | 94,90 | Butyl nitrite | 224,16 | 1 | 15,32 | 0,00342 | 7,10 |
| 15.32_224.1597m/z | CSID116 | 2M+NH4 | C4H9NO2 | -3,88 | 94,90 | gamma-Aminobutyric acid | 224,16 | 1 | 15,32 | 0,00342 | 7,10 |
| 15.32_224.1597m/z | CSID280126 | 2M+NH4 | C4H9NO2 | -3,88 | 94,90 | N-Ethylglycine | 224,16 | 1 | 15,32 | 0,00342 | 7,10 |
| 15.32_224.1597m/z | CSID388543 | 2M+NH4 | C4H9NO2 | -3,88 | 94,90 | S-beta-aminoisobutyric acid | 224,16 | 1 | 15,32 | 0,00342 | 7,10 |
| 15.32_224.1597m/z | CSID388757 | 2M+NH4 | C4H9NO2 | -3,88 | 94,90 | (R)-(âˆ’)-2-Aminobutyric acid | 224,16 | 1 | 15,32 | 0,00342 | 7,10 |
| 15.32_224.1597m/z | CSID4450824 | 2M+NH4 | C4H9NO2 | -3,88 | 94,90 | N-Methyl-L-alanine | 224,16 | 1 | 15,32 | 0,00342 | 7,10 |
| 15.32_224.1597m/z | CSID4573585 | 2M+NH4 | C4H9NO2 | -3,88 | 94,90 | R-BAIBA | 224,16 | 1 | 15,32 | 0,00342 | 7,10 |
| 15.32_224.1597m/z | CSID58481 | 2M+NH4 | C4H9NO2 | -3,88 | 94,90 | 3-Aminoisobutanoic acid | 224,16 | 1 | 15,32 | 0,00342 | 7,10 |
| 15.32_224.1597m/z | CSID5891 | 2M+NH4 | C4H9NO2 | -3,88 | 94,90 | 2-Aminoisobutyric Acid | 224,16 | 1 | 15,32 | 0,00342 | 7,10 |
| 15.32_224.1597m/z | CSID653 | 2M+NH4 | C4H9NO2 | -3,88 | 94,90 | N,N-Dimethylglycine | 224,16 | 1 | 15,32 | 0,00342 | 7,10 |
| 15.32_224.1597m/z | CSID72524 | 2M+NH4 | C4H9NO2 | -3,88 | 94,90 | (S)-(+)-2-Aminobutyric Acid | 224,16 | 1 | 15,32 | 0,00342 | 7,10 |
| 9.30_245.1347m/z | CSID21258154 | M+H | C12H20O5 | -14,98 | 90,79 | 6-Hydroxy-5-methyl-4,11-dioxoundecanoic acid | 245,13 | 1 | 9,30 | 0,00528 | 1,36 |
| 9.30_245.1347m/z | CSID35013400 | M+H-H2O | C12H22O6 | -13,93 | 90,55 | 5,8,12-Trihydroxy-2-oxododecanoic acid | 245,13 | 1 | 9,30 | 0,00528 | 1,36 |
| 16.83_314.1590m/z | CSID10298355 | 2M+ACN+H | C7H8N2O | -7,86 | 97,55 | 2-AB | 314,16 | 1 | 16,83 | 0,00890 | 217,60 |
| 16.83_314.1590m/z | CSID10302852 | 2M+ACN+H | C7H8N2O | -7,86 | 97,55 | 2-Methylnicotinamide | 314,16 | 1 | 16,83 | 0,00890 | 217,60 |
| 16.83_314.1590m/z | CSID29762 | 2M+ACN+H | C7H8N2O | -7,86 | 97,55 | 2-Acetyl-3-methylpyrazine | 314,16 | 1 | 16,83 | 0,00890 | 217,60 |
| 16.83_314.1590m/z | CSID35013654 | M+ACN+H | C9H16N6O4 | 6,94 | 93,30 | 4-(Hydroxymethyl)-2,6-diiminotetrahydro-1H,8H-pyrrolo[1,2-c]purine-5,10,10(6H,9H)-triol | 314,16 | 1 | 16,83 | 0,00890 | 217,60 |
| 16.83_314.1590m/z | CSID4515085 | 2M+ACN+H | C7H8N2O | -7,86 | 97,55 | 2-Methyl-4-acetylpyrimidine | 314,16 | 1 | 16,83 | 0,00890 | 217,60 |
| 16.83_314.1590m/z | CSID58476 | 2M+ACN+H | C7H8N2O | -7,86 | 97,55 | Nicotinyl methylamide | 314,16 | 1 | 16,83 | 0,00890 | 217,60 |
| 16.83_314.1590m/z | CSID5915 | 2M+ACN+H | C7H8N2O | -7,86 | 97,55 | YU0650000 | 314,16 | 1 | 16,83 | 0,00890 | 217,60 |
| 16.85_134.5813m/z | CSID112688 | M+H+Na | C12H23NO4 | 13,49 | 94,83 | 3-[(2,2-Dimethylpropanoyl)oxy]-4-(trimethylammonio)butanoate | 134,58 | 2 | 16,85 | 0,00958 | 38,24 |
| 16.85_134.5813m/z | CSID30776695 | M+H+Na | C12H23NO4 | 13,49 | 94,83 | (3S)-3-(Pentanoyloxy)-4-(trimethylammonio)butanoate | 134,58 | 2 | 16,85 | 0,00958 | 38,24 |
| 16.85_134.5813m/z | CSID4932271 | M+H+Na | C12H23NO4 | 13,49 | 94,83 | Isovalerylcarnitine | 134,58 | 2 | 16,85 | 0,00958 | 38,24 |
| 16.85_134.5813m/z | CSID4932320 | M+H+Na | C12H23NO4 | 13,49 | 94,83 | 2-methylbutyrylcarnitine | 134,58 | 2 | 16,85 | 0,00958 | 38,24 |
| 14.15_202.2150m/z | CSID10725083 | M+NH4 | C12H24O | -8,30 | 92,55 | 2,4,8-Trimethyl-7-nonen-2-ol | 202,22 | 1 | 14,15 | 0,01107 | 274,54 |
| 14.15_202.2150m/z | CSID21153 | M+NH4 | C12H24O | -8,30 | 92,55 | 2-Dodecanone | 202,22 | 1 | 14,15 | 0,01107 | 274,54 |
| 14.15_202.2150m/z | CSID54990 | M+NH4 | C12H24O | -8,30 | 92,55 | 2-Methylundecanal | 202,22 | 1 | 14,15 | 0,01107 | 274,54 |
| 14.15_202.2150m/z | CSID7902 | M+NH4 | C12H24O | -8,30 | 92,55 | Lauryl aldehyde | 202,22 | 1 | 14,15 | 0,01107 | 274,54 |
| 12.20_232.1897m/z | CSID388783 | M+NH4 | C12H22O3 | -4,91 | 92,51 | 3-oxolauric acid | 232,19 | 1 | 12,20 | 0,01129 | 6,08 |
| 12.20_232.1897m/z | CSID473391 | M+CH3OH+H | C11H21NO2 | -5,29 | 93,46 | 11-Nitro-1-undecene | 232,19 | 1 | 12,20 | 0,01129 | 6,08 |
| 12.20_232.1897m/z | CSID55379 | M+NH4 | C12H22O3 | -4,91 | 92,51 | 2-Hexyltetrahydrofuran-4-yl acetate | 232,19 | 1 | 12,20 | 0,01129 | 6,08 |
| 8.18_231.1197m/z | CSID10462 | 2M+Na | C5H12S | -7,04 | 94,31 | Isoamyl Mercaptan | 231,12 | 1 | 8,18 | 0,01291 | 1,77 |
| 8.18_231.1197m/z | CSID15090 | 2M+Na | C5H12S | -7,04 | 94,31 | 2-Methyl-1-butanethiol | 231,12 | 1 | 8,18 | 0,01291 | 1,77 |
| 8.18_231.1197m/z | CSID453431 | 2M+Na | C5H12S | -7,04 | 94,31 | 3-Methyl-2-butanethiol | 231,12 | 1 | 8,18 | 0,01291 | 1,77 |
| 8.18_231.1197m/z | CSID56209 | 2M+Na | C5H12S | -7,04 | 94,31 | 2-Pentanethiol | 231,12 | 1 | 8,18 | 0,01291 | 1,77 |
| 8.18_231.1197m/z | CSID7776 | 2M+Na | C5H12S | -7,04 | 94,31 | Pt | 231,12 | 1 | 8,18 | 0,01291 | 1,77 |
| 8.18_231.1197m/z | CSID81488 | M+Na | C9H20O5 | -2,86 | 93,91 | 3,6,9,12-tetraoxatridecan-1-ol | 231,12 | 1 | 8,18 | 0,01291 | 1,77 |
| 15.51_290.2683m/z | CSID10034 | M+NH4 | C16H32O3 | -2,26 | 95,21 | Juniperic acid | 290,27 | 1 | 15,51 | 0,02162 | 2,55 |
| 15.51_290.2683m/z | CSID10606337 | M+NH4 | C16H32O3 | -2,26 | 95,21 | 10-HYDROXYPALMITIC ACID | 290,27 | 1 | 15,51 | 0,02162 | 2,55 |
| 15.51_290.2683m/z | CSID13662332 | M+NH4 | C16H32O3 | -2,26 | 95,21 | 8-Hydroxyhexadecanoic acid | 290,27 | 1 | 15,51 | 0,02162 | 2,55 |
| 15.51_290.2683m/z | CSID13662333 | M+NH4 | C16H32O3 | -2,26 | 95,21 | 12-Hydroxyhexadecanoic acid | 290,27 | 1 | 15,51 | 0,02162 | 2,55 |
| 15.51_290.2683m/z | CSID13961377 | M+NH4 | C16H32O3 | -2,26 | 95,21 | 9-Hydroxyhexadecanoic acid | 290,27 | 1 | 15,51 | 0,02162 | 2,55 |
| 15.51_290.2683m/z | CSID21865352 | M+NH4 | C16H32O3 | -2,26 | 95,21 | (R)-3-hydroxypalmitic acid | 290,27 | 1 | 15,51 | 0,02162 | 2,55 |
| 15.51_290.2683m/z | CSID2282465 | M+NH4 | C16H32O3 | -2,26 | 95,21 | 11-Hydroxyhexadecanoic acid | 290,27 | 1 | 15,51 | 0,02162 | 2,55 |
| 15.51_290.2683m/z | CSID266538 | M+NH4 | C16H32O3 | -2,26 | 95,21 | MFCD00171638 | 290,27 | 1 | 15,51 | 0,02162 | 2,55 |
| 15.51_290.2683m/z | CSID4472186 | M+NH4 | C16H32O3 | -2,26 | 95,21 | 5-Hydroxyhexadecanoic acid | 290,27 | 1 | 15,51 | 0,02162 | 2,55 |
| 15.51_290.2683m/z | CSID74849809 | M+NH4 | C16H32O3 | -2,26 | 95,21 | 13-Hydroxyhexadecanoic acid | 290,27 | 1 | 15,51 | 0,02162 | 2,55 |
| 15.51_290.2683m/z | CSID83805 | M+NH4 | C16H32O3 | -2,26 | 95,21 | 2-Hydroxypalmitic Acid | 290,27 | 1 | 15,51 | 0,02162 | 2,55 |
| 15.51_290.2683m/z | CSID9240750 | M+NH4 | C16H32O3 | -2,26 | 95,21 | (R)-2-hydroxypalmitic acid | 290,27 | 1 | 15,51 | 0,02162 | 2,55 |
| 16.64_372.3101m/z | CSID110006 | M+ACN+H | C19H38O4 | -2,12 | 97,45 | MFCD00058513 | 372,31 | 1 | 16,64 | 0,02264 | 1,35 |
| 16.64_372.3101m/z | CSID14201 | M+ACN+H | C19H38O4 | -2,12 | 97,45 | L-Î±-PALMITIN | 372,31 | 1 | 16,64 | 0,02264 | 1,35 |
| 16.64_372.3101m/z | CSID2341519 | M+ACN+H | C19H38O4 | -2,12 | 97,45 | 1-palmitoyl-sn-glycerol | 372,31 | 1 | 16,64 | 0,02264 | 1,35 |
| 16.64_372.3101m/z | CSID30776567 | M+H | C21H41NO4 | -1,88 | 95,00 | C14-Carnitine | 372,31 | 1 | 16,64 | 0,02264 | 1,35 |
| 16.64_372.3101m/z | CSID417 | 2M+NH4 | C11H17N2+ | -2,66 | 93,70 | N-Methylnicotinium | 372,31 | 1 | 16,64 | 0,02264 | 1,35 |
| 16.64_372.3101m/z | CSID4517636 | M+NH4 | C21H38O4 | -1,97 | 95,38 | MFCD05863969 | 372,31 | 1 | 16,64 | 0,02264 | 1,35 |
| 16.64_372.3101m/z | CSID4941255 | M+NH4 | C21H38O4 | -1,97 | 95,38 | 1-linoleoyl-sn-glycerol | 372,31 | 1 | 16,64 | 0,02264 | 1,35 |
| 16.64_372.3101m/z | CSID4941514 | M+CH3OH+H | C20H37NO3 | -2,06 | 96,30 | MFCD01320532 | 372,31 | 1 | 16,64 | 0,02264 | 1,35 |
| 16.64_372.3101m/z | CSID59674321 | M+ACN+H | C19H38O4 | -2,12 | 97,45 | (2R)-2,3-Dihydroxypropyl 14-methylpentadecanoate | 372,31 | 1 | 16,64 | 0,02264 | 1,35 |
| 16.64_372.3101m/z | CSID59693720 | M+ACN+H | C19H38O4 | -2,12 | 97,45 | 1,3-Dihydroxy-2-propanyl 14-methylpentadecanoate | 372,31 | 1 | 16,64 | 0,02264 | 1,35 |
| 16.35_414.3201m/z | CSID30777162 | 2M+ACN+H | C14H18 | 12,37 | 98,45 | 5-Ethyl-3,8-dimethyl-1,7-dihydroazulene | 414,32 | 1 | 16,35 | 0,02900 | 1,44 |
| 16.35_414.3201m/z | CSID3395 | 2M+NH4 | C15H18 | 11,62 | 98,84 | guaiazulene | 414,32 | 1 | 16,35 | 0,02900 | 1,44 |
| 16.35_414.3201m/z | CSID34989566 | M+ACN+H | C21H40O5 | -3,37 | 91,29 | (2S)-3-Hydroxy-2-(octanoyloxy)propyl decanoate | 414,32 | 1 | 16,35 | 0,02900 | 1,44 |
| 16.35_414.3201m/z | CSID34989567 | M+ACN+H | C21H40O5 | -3,37 | 91,29 | (2R)-3-Hydroxy-2-(octanoyloxy)propyl decanoate | 414,32 | 1 | 16,35 | 0,02900 | 1,44 |
| 16.35_414.3201m/z | CSID34990996 | M+ACN+H | C21H40O5 | -3,37 | 91,29 | 2-Hydroxy-3-(octanoyloxy)propyl decanoate | 414,32 | 1 | 16,35 | 0,02900 | 1,44 |
| 16.35_414.3201m/z | CSID34990997 | M+ACN+H | C21H40O5 | -3,37 | 91,29 | 2-Hydroxy-3-(octanoyloxy)propyl decanoate | 414,32 | 1 | 16,35 | 0,02900 | 1,44 |
| 16.35_414.3201m/z | CSID34993673 | M+ACN+H | C21H40O5 | -3,37 | 91,29 | (2S)-1-Hydroxy-3-(octanoyloxy)-2-propanyl decanoate | 414,32 | 1 | 16,35 | 0,02900 | 1,44 |
| 16.35_414.3201m/z | CSID34993674 | M+ACN+H | C21H40O5 | -3,37 | 91,29 | (2R)-1-Hydroxy-3-(octanoyloxy)-2-propanyl decanoate | 414,32 | 1 | 16,35 | 0,02900 | 1,44 |
| 16.35_414.3201m/z | CSID35014013 | 2M+ACN+H | C14H18 | 12,37 | 98,45 | 7-Ethyl-1,4-dimethyl-4,5-dihydroazulene | 414,32 | 1 | 16,35 | 0,02900 | 1,44 |
| 16.35_414.3201m/z | CSID9808 | 2M+NH4 | C15H18 | 11,62 | 98,84 | cadalene | 414,32 | 1 | 16,35 | 0,02900 | 1,44 |
| 8.49_229.1035m/z | CSID26332128 | M+NH4 | C7H9N5O3 | -4,05 | 94,44 | 5,6,7,8-tetrahydropterin-6-carboxylic acid | 229,10 | 1 | 8,49 | 0,02937 | 1,69 |
| 15.51_230.2466m/z | CSID2286111 | M+NH4 | C14H28O | -5,77 | 92,71 | 12-Methyltridecanal | 230,25 | 1 | 15,51 | 0,03406 | 2,35 |
| 15.51_230.2466m/z | CSID29031 | M+NH4 | C14H28O | -5,77 | 92,71 | Myristaldehyde | 230,25 | 1 | 15,51 | 0,03406 | 2,35 |
| 15.51_230.2466m/z | CSID67900 | M+NH4 | C14H28O | -5,77 | 92,71 | 2-Tetradecanone | 230,25 | 1 | 15,51 | 0,03406 | 2,35 |
| 16.97_442.3521m/z | CSID143022 | M+ACN+H | C23H44O5 | -1,42 | 95,47 | (S)-1,2-didecanoylglycerol | 442,35 | 1 | 16,97 | 0,03523 | 1,55 |
| 16.97_442.3521m/z | CSID278038 | M+ACN+H | C23H44O5 | -1,42 | 95,47 | 1-ACETYL-3-MONOSTEARIN | 442,35 | 1 | 16,97 | 0,03523 | 1,55 |
| 16.97_442.3521m/z | CSID29899 | 2M+NH4 | C16H20 | 12,54 | 94,68 | 2,6-Di-iso-propylnaphthalene | 442,35 | 1 | 16,97 | 0,03523 | 1,55 |
| 16.97_442.3521m/z | CSID35032597 | M+H | C25H47NO5 | -1,29 | 93,07 | (4S)-4-{[(11Z)-3-Hydroxy-11-octadecenoyl]oxy}-4-(trimethylammonio)butanoate | 442,35 | 1 | 16,97 | 0,03523 | 1,55 |
| 16.97_442.3521m/z | CSID35032598 | M+H | C25H47NO5 | -1,29 | 93,07 | (4S)-4-{[(9Z)-3-Hydroxy-9-octadecenoyl]oxy}-4-(trimethylammonio)butanoate | 442,35 | 1 | 16,97 | 0,03523 | 1,55 |
| 16.97_442.3521m/z | CSID4474883 | 2M+ACN+H | C15H20 | 13,29 | 93,95 | 4-Isopropyl-1,6-dimethyl-1,2-dihydronaphthalene | 442,35 | 1 | 16,97 | 0,03523 | 1,55 |
| 16.97_442.3521m/z | CSID4475215 | 2M+ACN+H | C15H20 | 13,29 | 93,95 | 5-Isopropyl-3,8-dimethyl-1,2-dihydronaphthalene | 442,35 | 1 | 16,97 | 0,03523 | 1,55 |
| 16.97_442.3521m/z | CSID460799 | 2M+ACN+H | C15H20 | 13,29 | 93,95 | 3,4-Dihydrocadalene | 442,35 | 1 | 16,97 | 0,03523 | 1,55 |
| 16.97_442.3521m/z | CSID461487 | 2M+ACN+H | C15H20 | 13,29 | 93,95 | 4-Isopropyl-6-methyl-1-methylene-1,2,3,4-tetrahydronaphthalene | 442,35 | 1 | 16,97 | 0,03523 | 1,55 |
| 16.97_442.3521m/z | CSID511806 | 2M+ACN+H | C15H20 | 13,29 | 93,95 | Isolongifolene, 4,5,9,10-dehydro- | 442,35 | 1 | 16,97 | 0,03523 | 1,55 |
| 16.97_442.3521m/z | CSID59692101 | M+ACN+H | C23H44O5 | -1,42 | 95,47 | (2R)-3-Hydroxy-2-(octanoyloxy)propyl laurate | 442,35 | 1 | 16,97 | 0,03523 | 1,55 |
| 16.97_442.3521m/z | CSID59692102 | M+ACN+H | C23H44O5 | -1,42 | 95,47 | 2-Hydroxy-3-(octanoyloxy)propyl laurate | 442,35 | 1 | 16,97 | 0,03523 | 1,55 |
| 16.97_442.3521m/z | CSID59692103 | M+ACN+H | C23H44O5 | -1,42 | 95,47 | (2R)-3-Hydroxy-2-(octanoyloxy)propyl 10-methylundecanoate | 442,35 | 1 | 16,97 | 0,03523 | 1,55 |
| 16.97_442.3521m/z | CSID59692104 | M+ACN+H | C23H44O5 | -1,42 | 95,47 | 2-Hydroxy-3-(octanoyloxy)propyl 10-methylundecanoate | 442,35 | 1 | 16,97 | 0,03523 | 1,55 |
| 16.97_442.3521m/z | CSID59694815 | M+ACN+H | C23H44O5 | -1,42 | 95,47 | (2R)-1-Hydroxy-3-(octanoyloxy)-2-propanyl laurate | 442,35 | 1 | 16,97 | 0,03523 | 1,55 |
| 16.97_442.3521m/z | CSID59694816 | M+ACN+H | C23H44O5 | -1,42 | 95,47 | 2-Hydroxy-3-(octanoyloxy)propyl laurate | 442,35 | 1 | 16,97 | 0,03523 | 1,55 |
| 16.97_442.3521m/z | CSID59694817 | M+ACN+H | C23H44O5 | -1,42 | 95,47 | (2R)-1-Hydroxy-3-(octanoyloxy)-2-propanyl 10-methylundecanoate | 442,35 | 1 | 16,97 | 0,03523 | 1,55 |
| 16.97_442.3521m/z | CSID59694818 | M+ACN+H | C23H44O5 | -1,42 | 95,47 | 2-Hydroxy-3-(octanoyloxy)propyl 10-methylundecanoate | 442,35 | 1 | 16,97 | 0,03523 | 1,55 |
| 16.97_442.3521m/z | CSID74857958 | M+ACN+H | C23H44O5 | -1,42 | 95,47 | (2S)-1-Hydroxy-3-(octanoyloxy)-2-propanyl laurate | 442,35 | 1 | 16,97 | 0,03523 | 1,55 |
| 16.97_442.3521m/z | CSID74857959 | M+ACN+H | C23H44O5 | -1,42 | 95,47 | (2S)-1-Hydroxy-3-(octanoyloxy)-2-propanyl 10-methylundecanoate | 442,35 | 1 | 16,97 | 0,03523 | 1,55 |
| 16.97_442.3521m/z | CSID74858013 | M+ACN+H | C23H44O5 | -1,42 | 95,47 | (2S)-3-Hydroxy-2-(octanoyloxy)propyl laurate | 442,35 | 1 | 16,97 | 0,03523 | 1,55 |
| 16.97_442.3521m/z | CSID74858014 | M+ACN+H | C23H44O5 | -1,42 | 95,47 | (2S)-3-Hydroxy-2-(octanoyloxy)propyl 10-methylundecanoate | 442,35 | 1 | 16,97 | 0,03523 | 1,55 |
| 16.97_442.3521m/z | CSID9010161 | M+ACN+H | C23H44O5 | -1,42 | 95,47 | (R)-1,2-didecanoylglycerol | 442,35 | 1 | 16,97 | 0,03523 | 1,55 |
| 16.97_442.3521m/z | CSID94487 | M+ACN+H | C23H44O5 | -1,42 | 95,47 | Didecanoin | 442,35 | 1 | 16,97 | 0,03523 | 1,55 |
| 16.97_386.3252m/z | CSID107267 | 2M+ACN+H | C10H20O2 | -3,73 | 97,73 | 3,7-Dimethyl-1-octen-3,7-diol | 386,33 | 1 | 16,97 | 0,03776 | 1,51 |
| 16.97_386.3252m/z | CSID10771160 | 2M+NH4 | C11H20O2 | -3,48 | 96,69 | 10-Undecenoic acid | 386,33 | 1 | 16,97 | 0,03776 | 1,51 |
| 16.97_386.3252m/z | CSID11791 | 2M+ACN+H | C10H20O2 | -3,73 | 97,73 | MO6950000 | 386,33 | 1 | 16,97 | 0,03776 | 1,51 |
| 16.97_386.3252m/z | CSID12093 | 2M+ACN+H | C10H20O2 | -3,73 | 97,73 | Isoamyl isovalerate | 386,33 | 1 | 16,97 | 0,03776 | 1,51 |
| 16.97_386.3252m/z | CSID148120 | 2M+NH4 | C11H20O2 | -3,48 | 96,69 | 3-Acetoxy-1-nonene | 386,33 | 1 | 16,97 | 0,03776 | 1,51 |
| 16.97_386.3252m/z | CSID14846 | 2M+ACN+H | C10H20O2 | -3,73 | 97,73 | RA6906000 | 386,33 | 1 | 16,97 | 0,03776 | 1,51 |
| 16.97_386.3252m/z | CSID151463 | 2M+NH4 | C11H20O2 | -3,48 | 96,69 | Prenyl caproate | 386,33 | 1 | 16,97 | 0,03776 | 1,51 |
| 16.97_386.3252m/z | CSID15988 | 2M+ACN+H | C10H20O2 | -3,73 | 97,73 | NQ4695000 | 386,33 | 1 | 16,97 | 0,03776 | 1,51 |
| 16.97_386.3252m/z | CSID16213 | 2M+ACN+H | C10H20O2 | -3,73 | 97,73 | MFCD00059395 | 386,33 | 1 | 16,97 | 0,03776 | 1,51 |
| 16.97_386.3252m/z | CSID16570 | 2M+ACN+H | C10H20O2 | -3,73 | 97,73 | ET4203000 | 386,33 | 1 | 16,97 | 0,03776 | 1,51 |
| 16.97_386.3252m/z | CSID19046 | 2M+NH4 | C11H20O2 | -3,48 | 96,69 | RH0365000 | 386,33 | 1 | 16,97 | 0,03776 | 1,51 |
| 16.97_386.3252m/z | CSID19175 | 2M+ACN+H | C10H20O2 | -3,73 | 97,73 | MFCD01730120 | 386,33 | 1 | 16,97 | 0,03776 | 1,51 |
| 16.97_386.3252m/z | CSID199694 | 2M+ACN+H | C10H20O2 | -3,73 | 97,73 | Propyl heptanoate | 386,33 | 1 | 16,97 | 0,03776 | 1,51 |
| 16.97_386.3252m/z | CSID20138479 | 2M+NH4 | C11H20O2 | -3,48 | 96,69 | PEARLATE | 386,33 | 1 | 16,97 | 0,03776 | 1,51 |
| 16.97_386.3252m/z | CSID251275 | 2M+NH4 | C11H20O2 | -3,48 | 96,69 | 4-Methyl-4-decanolide | 386,33 | 1 | 16,97 | 0,03776 | 1,51 |
| 16.97_386.3252m/z | CSID253449 | 2M+NH4 | C11H20O2 | -3,48 | 96,69 | MFCD00046351 | 386,33 | 1 | 16,97 | 0,03776 | 1,51 |
| 16.97_386.3252m/z | CSID2863 | 2M+ACN+H | C10H20O2 | -3,73 | 97,73 | Decanoic acid | 386,33 | 1 | 16,97 | 0,03776 | 1,51 |
| 16.97_386.3252m/z | CSID30776879 | 2M+NH4 | C11H20O2 | -3,48 | 96,69 | (3Z)-3-Methyl-3-decenoic acid | 386,33 | 1 | 16,97 | 0,03776 | 1,51 |
| 16.97_386.3252m/z | CSID30776896 | 2M+NH4 | C11H20O2 | -3,48 | 96,69 | Ethyl (2Z)-2-nonenoate | 386,33 | 1 | 16,97 | 0,03776 | 1,51 |
| 16.97_386.3252m/z | CSID35013311 | 2M+NH4 | C11H20O2 | -3,48 | 96,69 | (4Z)-3-Methyl-4-decenoic acid | 386,33 | 1 | 16,97 | 0,03776 | 1,51 |
| 16.97_386.3252m/z | CSID4509339 | 2M+NH4 | C11H20O2 | -3,48 | 96,69 | (2E)-Hex-2-enyl 2-methylbutyrate | 386,33 | 1 | 16,97 | 0,03776 | 1,51 |
| 16.97_386.3252m/z | CSID4509340 | 2M+NH4 | C11H20O2 | -3,48 | 96,69 | trans-2-Hexenyl Isovalerate | 386,33 | 1 | 16,97 | 0,03776 | 1,51 |
| 16.97_386.3252m/z | CSID4509760 | 2M+NH4 | C11H20O2 | -3,48 | 96,69 | (2E)-2-Hexenyl pentanoate | 386,33 | 1 | 16,97 | 0,03776 | 1,51 |
| 16.97_386.3252m/z | CSID4515527 | 2M+NH4 | C11H20O2 | -3,48 | 96,69 | (2Z)-2-Pentenyl hexanoate | 386,33 | 1 | 16,97 | 0,03776 | 1,51 |
| 16.97_386.3252m/z | CSID4517162 | 2M+NH4 | C11H20O2 | -3,48 | 96,69 | MFCD00036530 | 386,33 | 1 | 16,97 | 0,03776 | 1,51 |
| 16.97_386.3252m/z | CSID4517218 | 2M+ACN+H | C10H20O2 | -3,73 | 97,73 | 1,1-diethoxyhex-2-ene | 386,33 | 1 | 16,97 | 0,03776 | 1,51 |
| 16.97_386.3252m/z | CSID4519169 | 2M+NH4 | C11H20O2 | -3,48 | 96,69 | (3Z)-Hex-3-en-1-yl isovalerate | 386,33 | 1 | 16,97 | 0,03776 | 1,51 |
| 16.97_386.3252m/z | CSID4519170 | 2M+NH4 | C11H20O2 | -3,48 | 96,69 | 3-Hexenyl pentanoate | 386,33 | 1 | 16,97 | 0,03776 | 1,51 |
| 16.97_386.3252m/z | CSID4519468 | 2M+NH4 | C11H20O2 | -3,48 | 96,69 | Methyl decenoate | 386,33 | 1 | 16,97 | 0,03776 | 1,51 |
| 16.97_386.3252m/z | CSID45359 | 2M+ACN+H | C10H20O2 | -3,73 | 97,73 | 6-Methyl-2-heptanyl acetate | 386,33 | 1 | 16,97 | 0,03776 | 1,51 |
| 16.97_386.3252m/z | CSID453868 | 2M+ACN+H | C10H20O2 | -3,73 | 97,73 | MFCD00048645 | 386,33 | 1 | 16,97 | 0,03776 | 1,51 |
| 16.97_386.3252m/z | CSID454194 | 2M+ACN+H | C10H20O2 | -3,73 | 97,73 | Isopropyl heptanoate | 386,33 | 1 | 16,97 | 0,03776 | 1,51 |
| 16.97_386.3252m/z | CSID454668 | 2M+ACN+H | C10H20O2 | -3,73 | 97,73 | 3-Octanyl acetate | 386,33 | 1 | 16,97 | 0,03776 | 1,51 |
| 16.97_386.3252m/z | CSID456980 | 2M+ACN+H | C10H20O2 | -3,73 | 97,73 | p-Menthane-1,3-diol | 386,33 | 1 | 16,97 | 0,03776 | 1,51 |
| 16.97_386.3252m/z | CSID4576467 | 2M+ACN+H | C10H20O2 | -3,73 | 97,73 | (E)-1-(1-Ethoxyethoxy)-3-hexene | 386,33 | 1 | 16,97 | 0,03776 | 1,51 |
| 16.97_386.3252m/z | CSID473227 | 2M+NH4 | C11H20O2 | -3,48 | 96,69 | (2-Hexylcyclopropyl)acetic acid | 386,33 | 1 | 16,97 | 0,03776 | 1,51 |
| 16.97_386.3252m/z | CSID4829022 | 2M+NH4 | C11H20O2 | -3,48 | 96,69 | 3-Hexenyl 2-methylbutyrate | 386,33 | 1 | 16,97 | 0,03776 | 1,51 |
| 16.97_386.3252m/z | CSID484204 | 2M+ACN+H | C10H20O2 | -3,73 | 97,73 | 2-(2-Hydroxy-2-propanyl)-5-methylcyclohexanol | 386,33 | 1 | 16,97 | 0,03776 | 1,51 |
| 16.97_386.3252m/z | CSID4941719 | 2M+NH4 | C11H20O2 | -3,48 | 96,69 | trans-3-Heptenyl isobutyrate | 386,33 | 1 | 16,97 | 0,03776 | 1,51 |
| 16.97_386.3252m/z | CSID55044 | 2M+NH4 | C11H20O2 | -3,48 | 96,69 | Rhodinyl formate | 386,33 | 1 | 16,97 | 0,03776 | 1,51 |
| 16.97_386.3252m/z | CSID55148 | 2M+NH4 | C11H20O2 | -3,48 | 96,69 | UQ1320000 | 386,33 | 1 | 16,97 | 0,03776 | 1,51 |
| 16.97_386.3252m/z | CSID55229 | 2M+NH4 | C11H20O2 | -3,48 | 96,69 | Methyl rhodinolate | 386,33 | 1 | 16,97 | 0,03776 | 1,51 |
| 16.97_386.3252m/z | CSID55333 | 2M+NH4 | C11H20O2 | -3,48 | 96,69 | 25ODI8S42W | 386,33 | 1 | 16,97 | 0,03776 | 1,51 |
| 16.97_386.3252m/z | CSID55387 | 2M+NH4 | C11H20O2 | -3,48 | 96,69 | MFCD00001526 | 386,33 | 1 | 16,97 | 0,03776 | 1,51 |
| 16.97_386.3252m/z | CSID55713 | 2M+ACN+H | C10H20O2 | -3,73 | 97,73 | 4-Ethylcaprylic acid | 386,33 | 1 | 16,97 | 0,03776 | 1,51 |
| 16.97_386.3252m/z | CSID55851 | 2M+ACN+H | C10H20O2 | -3,73 | 97,73 | 4-Methylnonanoic acid | 386,33 | 1 | 16,97 | 0,03776 | 1,51 |
| 16.97_386.3252m/z | CSID56226 | 2M+ACN+H | C10H20O2 | -3,73 | 97,73 | FEMA 3506 | 386,33 | 1 | 16,97 | 0,03776 | 1,51 |
| 16.97_386.3252m/z | CSID6399 | 2M+ACN+H | C10H20O2 | -3,73 | 97,73 | Terpin | 386,33 | 1 | 16,97 | 0,03776 | 1,51 |
| 16.97_386.3252m/z | CSID66412 | 2M+NH4 | C11H20O2 | -3,48 | 96,69 | Cyclohexyl valerate | 386,33 | 1 | 16,97 | 0,03776 | 1,51 |
| 16.97_386.3252m/z | CSID67464 | 2M+ACN+H | C10H20O2 | -3,73 | 97,73 | Isoamyl valerate | 386,33 | 1 | 16,97 | 0,03776 | 1,51 |
| 16.97_386.3252m/z | CSID7428 | 2M+NH4 | C11H20O2 | -3,48 | 96,69 | YQ2485000 | 386,33 | 1 | 16,97 | 0,03776 | 1,51 |
| 16.97_386.3252m/z | CSID7487 | 2M+ACN+H | C10H20O2 | -3,73 | 97,73 | MO8389000 | 386,33 | 1 | 16,97 | 0,03776 | 1,51 |
| 16.97_386.3252m/z | CSID7490 | 2M+NH4 | C11H20O2 | -3,48 | 96,69 | RH3480000 | 386,33 | 1 | 16,97 | 0,03776 | 1,51 |
| 16.97_386.3252m/z | CSID7511 | 2M+ACN+H | C10H20O2 | -3,73 | 97,73 | RH0680000 | 386,33 | 1 | 16,97 | 0,03776 | 1,51 |
| 16.97_386.3252m/z | CSID7600 | 2M+ACN+H | C10H20O2 | -3,73 | 97,73 | RG7850000 | 386,33 | 1 | 16,97 | 0,03776 | 1,51 |
| 16.97_386.3252m/z | CSID7872 | 2M+ACN+H | C10H20O2 | -3,73 | 97,73 | AJ1400000 | 386,33 | 1 | 16,97 | 0,03776 | 1,51 |
| 16.97_386.3252m/z | CSID86646 | 2M+ACN+H | C10H20O2 | -3,73 | 97,73 | Amyl isovalerate | 386,33 | 1 | 16,97 | 0,03776 | 1,51 |
| 16.97_386.3252m/z | CSID8830141 | 2M+ACN+H | C10H20O2 | -3,73 | 97,73 | (+)-2-ethylhexyl acetate | 386,33 | 1 | 16,97 | 0,03776 | 1,51 |
| 16.97_386.3252m/z | CSID9216580 | 2M+NH4 | C11H20O2 | -3,48 | 96,69 | cis-5-Octenyl propionate | 386,33 | 1 | 16,97 | 0,03776 | 1,51 |
| 16.97_386.3252m/z | CSID9259492 | 2M+ACN+H | C10H20O2 | -3,73 | 97,73 | 4,5-Dimethyl-2-pentyl-1,3-dioxolane | 386,33 | 1 | 16,97 | 0,03776 | 1,51 |
| 16.97_386.3252m/z | CSID9280962 | 2M+ACN+H | C10H20O2 | -3,73 | 97,73 | 2-Methyl-4-pentyl-1,3-dioxane | 386,33 | 1 | 16,97 | 0,03776 | 1,51 |
| 16.97_386.3252m/z | CSID92970 | 2M+ACN+H | C10H20O2 | -3,73 | 97,73 | Ethyl 2-ethylcaproate | 386,33 | 1 | 16,97 | 0,03776 | 1,51 |
| 16.97_386.3252m/z | CSID96085 | 2M+NH4 | C11H20O2 | -3,48 | 96,69 | ÃŽÂ²-Methyl-ÃŽÂ³-decalactone | 386,33 | 1 | 16,97 | 0,03776 | 1,51 |
| 16.97_386.3252m/z | CSID96446 | 2M+ACN+H | C10H20O2 | -3,73 | 97,73 | cis-2-Hydroxymenthol | 386,33 | 1 | 16,97 | 0,03776 | 1,51 |
| 16.97_386.3252m/z | CSID98237 | 2M+NH4 | C11H20O2 | -3,48 | 96,69 | 2,4-Dimethyl-2-(4-methylpent-3-en-1-yl)-1,3-dioxolane | 386,33 | 1 | 16,97 | 0,03776 | 1,51 |
| 16.63_411.0281m/z | CSID10208 | 2M+K | C11H6O3 | 4,24 | 94,26 | Angelicin | 411,03 | 1 | 16,63 | 0,04534 | 1,13 |
| 16.63_411.0281m/z | CSID10469099 | 2M+Na | C9H6O5 | -10,72 | 95,77 | 1,3-Benzodioxol-5-yl(oxo)acetic acid | 411,03 | 1 | 16,63 | 0,04534 | 1,13 |
| 16.63_411.0281m/z | CSID10523336 | 2M+Na | C9H6O5 | -10,72 | 95,77 | 4,5,7-Trihydroxycoumarin | 411,03 | 1 | 16,63 | 0,04534 | 1,13 |
| 16.63_411.0281m/z | CSID145893 | M+2Na-H | C10H15N4O9P | -1,97 | 92,39 | 5-Formamidoimidazole-4-carboxamide ribotide | 411,03 | 1 | 16,63 | 0,04534 | 1,13 |
| 16.63_411.0281m/z | CSID32682270 | 2M+Na | C9H6O5 | -10,72 | 95,77 | 5,6,7-Trihydroxy-2H-chromen-2-one | 411,03 | 1 | 16,63 | 0,04534 | 1,13 |
| 16.63_411.0281m/z | CSID5964 | 2M+K | C11H6O3 | 4,24 | 94,26 | Psoralen | 411,03 | 1 | 16,63 | 0,04534 | 1,13 |
| 16.63_411.0281m/z | CSID60828098 | 2M+Na | C9H6O5 | -10,72 | 95,77 | 4,6,7-Trihydroxy-2H-chromen-2-one | 411,03 | 1 | 16,63 | 0,04534 | 1,13 |
| 16.63_411.0281m/z | CSID9994589 | 2M+Na | C9H6O5 | -10,72 | 95,77 | 6,7,8-Trihydroxy-2H-chromen-2-one | 411,03 | 1 | 16,63 | 0,04534 | 1,13 |
| 16.85_398.3249m/z | CSID23010051 | M+ACN+H | C21H40O4 | -4,44 | 91,80 | 1-Oleoyl-sn-glycerol | 398,32 | 1 | 16,85 | 0,04861 | 1,35 |
| 16.85_398.3249m/z | CSID24765770 | M+ACN+H | C21H40O4 | -4,44 | 91,80 | 1,3-Dihydroxy-2-propanyl (11Z)-11-octadecenoate | 398,32 | 1 | 16,85 | 0,04861 | 1,35 |
| 16.85_398.3249m/z | CSID24765787 | M+ACN+H | C21H40O4 | -4,44 | 91,80 | (2S)-2,3-Dihydroxypropyl (11Z)-11-octadecenoate | 398,32 | 1 | 16,85 | 0,04861 | 1,35 |
| 16.85_398.3249m/z | CSID35013688 | M+ACN+H | C21H40O4 | -4,44 | 91,80 | 3-[(2E)-2-Hepten-1-yloxy]-2-hydroxypropyl undecanoate | 398,32 | 1 | 16,85 | 0,04861 | 1,35 |
| 16.85_398.3249m/z | CSID4446588 | M+ACN+H | C21H40O4 | -4,44 | 91,80 | 1-Oleoyl-rac-glycerol | 398,32 | 1 | 16,85 | 0,04861 | 1,35 |
| 16.85_398.3249m/z | CSID4478086 | M+ACN+H | C21H40O4 | -4,44 | 91,80 | 2-Oleoylglycerol | 398,32 | 1 | 16,85 | 0,04861 | 1,35 |
| 16.85_398.3249m/z | CSID4942643 | M+CH3OH+H | C22H39NO3 | -4,33 | 90,72 | N-LINOLEOYL-4-AMINOBUTYRIC ACID | 398,32 | 1 | 16,85 | 0,04861 | 1,35 |
| 16.85_398.3249m/z | CSID78181 | M+ACN+H | C21H40O4 | -4,44 | 91,80 | Stearoyllactic acid | 398,32 | 1 | 16,85 | 0,04861 | 1,35 |
| 16.78_416.3363m/z | CSID28639186 | M+H | C23H45NO5 | -1,86 | 93,57 | 3-hydroxyhexadecanoylcarnitine | 416,34 | 1 | 16,78 | 0,04977 | 1,34 |
| 16.78_416.3363m/z | CSID35032595 | M+H | C23H45NO5 | -1,86 | 93,57 | (4S)-4-[(3-Hydroxyhexadecanoyl)oxy]-4-(trimethylammonio)butanoate | 416,34 | 1 | 16,78 | 0,04977 | 1,34 |
| 16.78_416.3363m/z | CSID35032596 | M+H | C23H45NO5 | -1,86 | 93,57 | (4S)-4-[(2-Hydroxyhexadecanoyl)oxy]-4-(trimethylammonio)butanoate | 416,34 | 1 | 16,78 | 0,04977 | 1,34 |

Supplementary Table 2: Other compounds identified as significantly (p<0.05; ANOVA) changed upon exposure to cUMP in the untargeted mass spectrometric screening (negative ionization mode)

| **Compound** | **Compound ID** | **Adducts** | **Formula** | **Mass Error (ppm)** | **Isotope Similarity** | **Description** | **m/z** | **Charge** | **Retention time (min)** | **Anova (p)** | **Max Fold Change** |
| --- | --- | --- | --- | --- | --- | --- | --- | --- | --- | --- | --- |
| 14.86_242.1756m/z | CSID399857 | M-H | C13H25NO3 | -2,51 | 95,67 | N-Undecanoylglycine | 242,175554 | 1 | 14,86 | 0,001 | 1,60 |
| 14.86_372.1318m/z | CSID10443429 | M+Na-2H | C20H21N3O3 | -3,31 | 92,06 | Phe-Trp | 372,131799 | 1 | 14,86 | 0,009 | 2,17 |
| 14.86_372.1318m/z | CSID3627800 | M+Na-2H | C20H21N3O3 | -3,31 | 92,06 | Trp-Phe | 372,131799 | 1 | 14,86 | 0,009 | 2,17 |
| 1.25_242.0782m/z | CSID240418 | M+FA-H | C8H11N3O3 | -0,41 | 90,16 | N-Acetyl-DL-histidine | 242,07817 | 1 | 1,25 | 0,012 | 1,43 |
| 1.25_242.0782m/z | CSID390074 | M+Cl | C8H17NO5 | -9,19 | 90,27 | Miglitol | 242,07817 | 1 | 1,25 | 0,012 | 1,43 |
| 15.61_337.2055m/z | CSID388530 | 2M+FA-H | C6H14N2O2 | -12,82 | 98,44 | L-beta-lysine | 337,205517 | 1 | 15,61 | 0,024 | 1,41 |
| 15.61_337.2055m/z | CSID388540 | 2M+FA-H | C6H14N2O2 | -12,82 | 98,44 | (3S,5S)-3,5-Diaminohexanoic acid | 337,205517 | 1 | 15,61 | 0,024 | 1,41 |
| 15.61_337.2055m/z | CSID51793 | 2M+FA-H | C6H14N2O2 | -12,82 | 98,44 | D-Lysine | 337,205517 | 1 | 15,61 | 0,024 | 1,41 |
| 15.61_337.2055m/z | CSID5747 | 2M+FA-H | C6H14N2O2 | -12,82 | 98,44 | L-(+)-Lysine | 337,205517 | 1 | 15,61 | 0,024 | 1,41 |
| 15.61_337.2055m/z | CSID843 | 2M+FA-H | C6H14N2O2 | -12,82 | 98,44 | DL-Lysine | 337,205517 | 1 | 15,61 | 0,024 | 1,41 |
