# Supplementary material for: The effect of cCMP and cUMP on growth of Pseudomonas aeruginosa
Source: Front Microbiol. 2025 Sep 16;16:1675794. doi: 10.3389/fmicb.2025.1675794 (PMC12489823; doi:10.3389/fmicb.2025.1675794)
Supplement: Supplementary file 2 [file Presentation_1.pptx]

## Slide 1
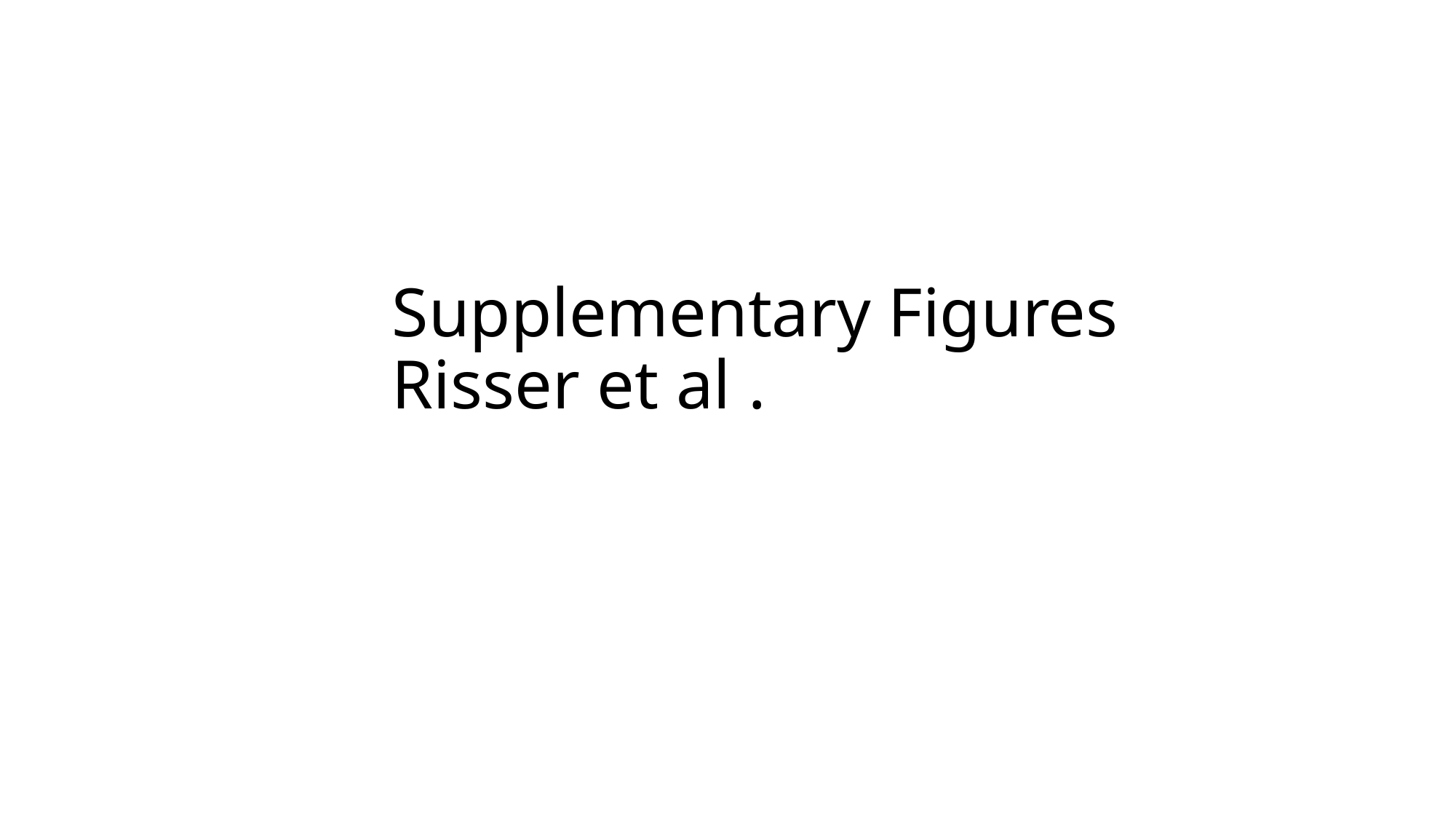

Supplementary Figures
Risser et al .

## Slide 2
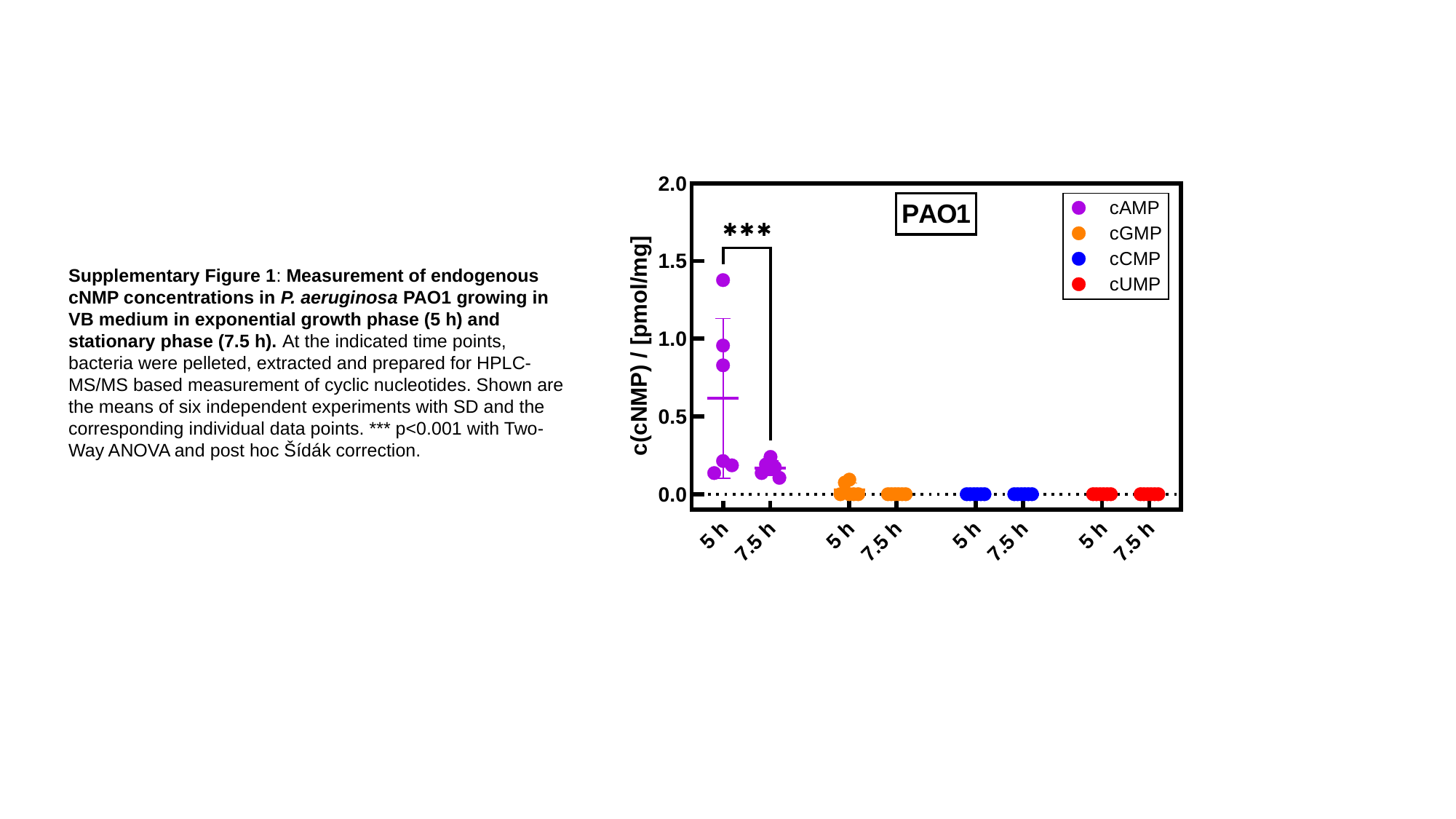

Supplementary Figure 1: Measurement of endogenous cNMP concentrations in P. aeruginosa PAO1 growing in VB medium in exponential growth phase (5 h) and stationary phase (7.5 h). At the indicated time points, bacteria were pelleted, extracted and prepared for HPLC-MS/MS based measurement of cyclic nucleotides. Shown are the means of six independent experiments with SD and the corresponding individual data points. *** p<0.001 with Two-Way ANOVA and post hoc Šídák correction.

## Slide 3
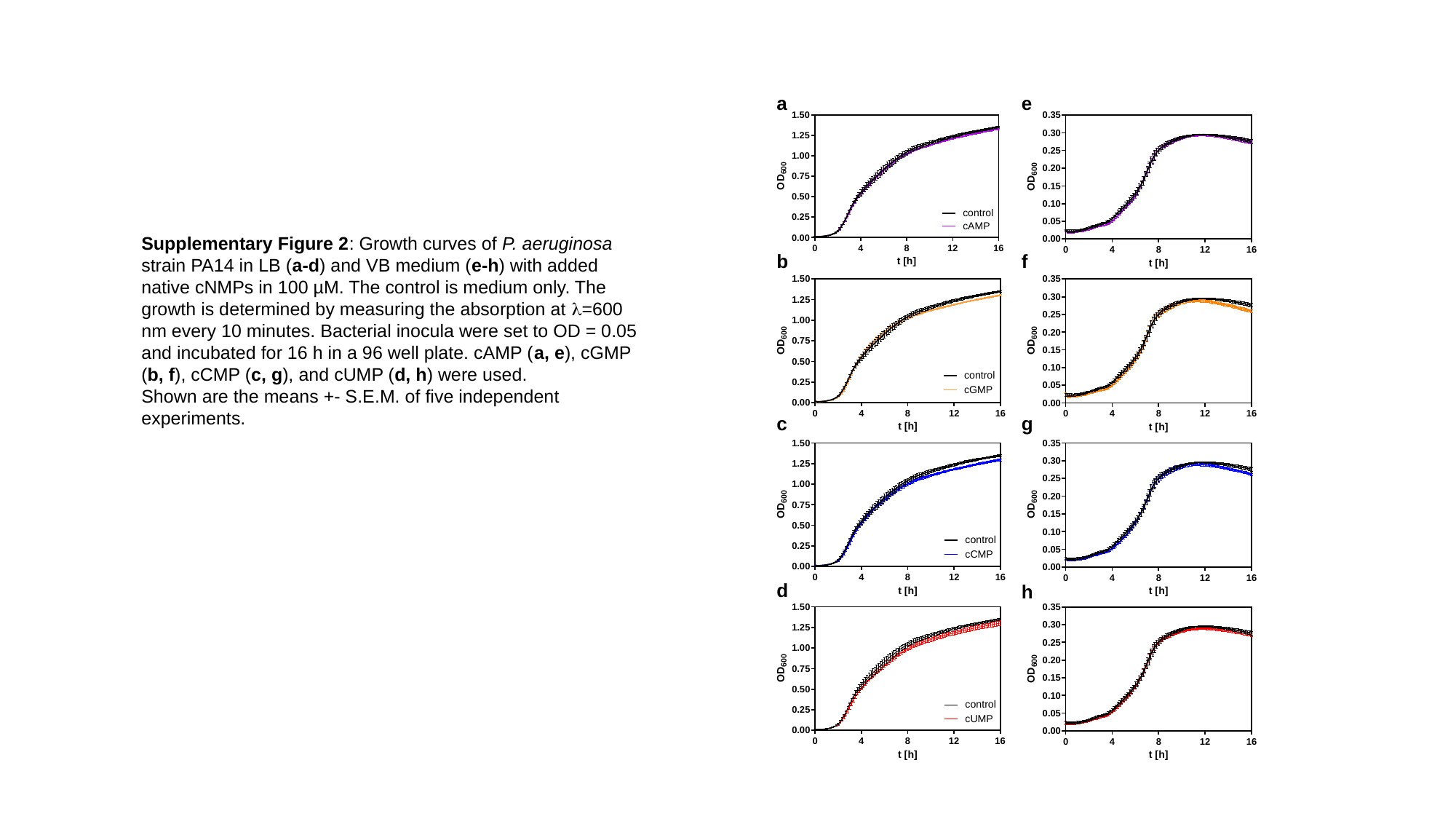

Supplementary Figure 2: Growth curves of P. aeruginosa strain PA14 in LB (a-d) and VB medium (e-h) with added native cNMPs in 100 µM. The control is medium only. The growth is determined by measuring the absorption at l=600 nm every 10 minutes. Bacterial inocula were set to OD = 0.05 and incubated for 16 h in a 96 well plate. cAMP (a, e), cGMP (b, f), cCMP (c, g), and cUMP (d, h) were used.
Shown are the means +- S.E.M. of five independent experiments.

## Slide 4
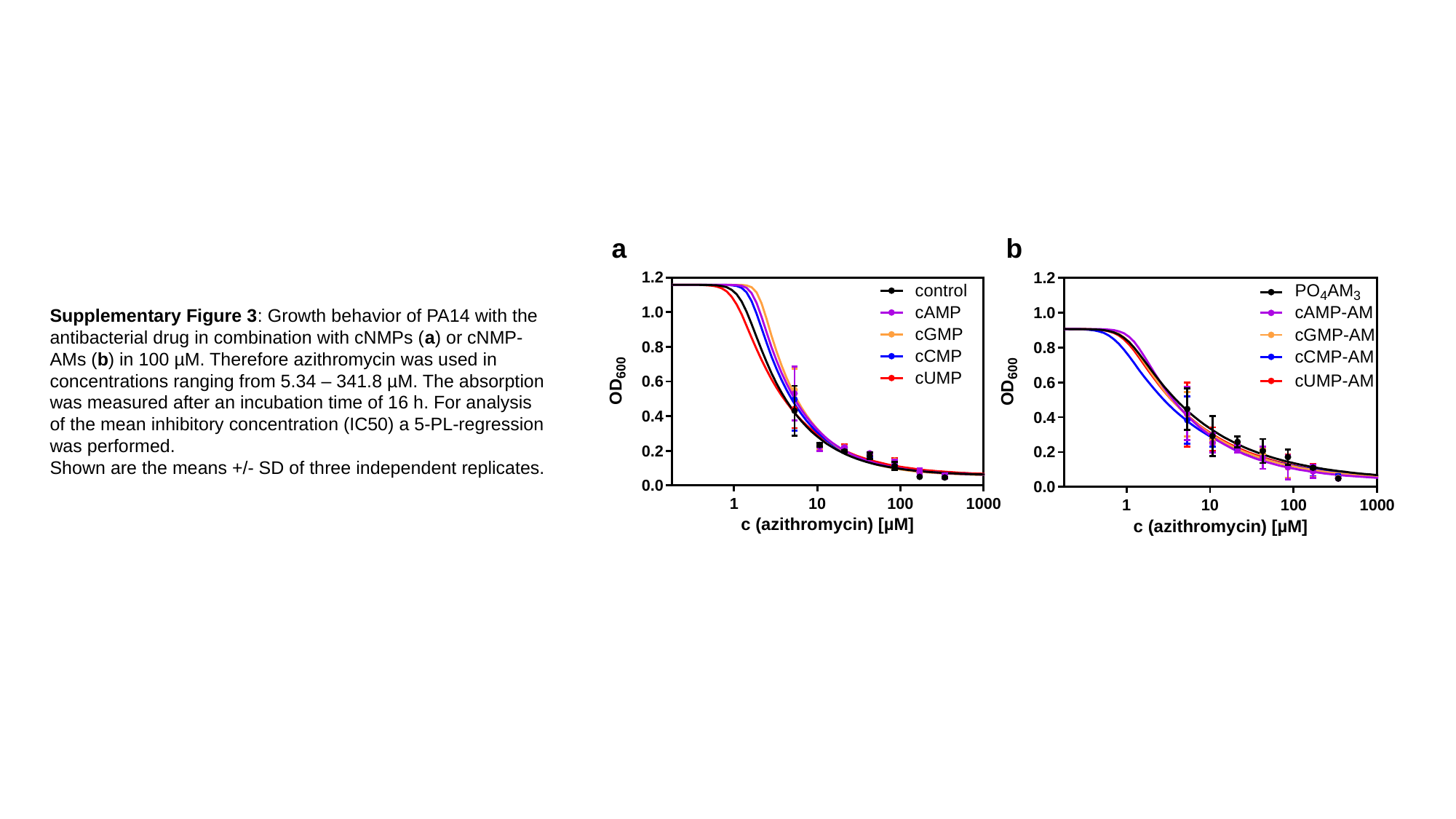

Supplementary Figure 3: Growth behavior of PA14 with the antibacterial drug in combination with cNMPs (a) or cNMP-AMs (b) in 100 µM. Therefore azithromycin was used in concentrations ranging from 5.34 – 341.8 µM. The absorption was measured after an incubation time of 16 h. For analysis of the mean inhibitory concentration (IC50) a 5-PL-regression was performed.
Shown are the means +/- SD of three independent replicates.
